# The viral expression and immune status in human cancers and insights into novel biomarkers of immunotherapy

**DOI:** 10.1186/s12885-021-08871-9

**Published:** 2021-11-05

**Authors:** Siyuan Chen, Hongyan Lai, Jingjing Zhao, Bing Chen, Yan Li, Yuchen Li, Qin Li, Qiupeng Zheng, Shenglin Huang, Xiaodong Zhu

**Affiliations:** 1 Department of Medical Oncology, Shanghai Key Laboratory of Medical Epigenetics, Fudan University Shanghai Cancer Center, Institutes of Biomedical Sciences, Fudan University, 270 Dong An Rd, Shanghai, 200032 China; 2 Department of Oncology, Shanghai Medical College, Fudan University, Shanghai, China

**Keywords:** Viral infections, Tumor immune microenvironment (TIME), Immunotherapy, Machine learning, pan-cancer analysis

## Abstract

**Background:**

Viral infections are prevalent in human cancers and they have great diagnostic and theranostic values in clinical practice. Recently, their potential of shaping the tumor immune microenvironment (TIME) has been related to the immunotherapy of human cancers. However, the landscape of viral expressions and immune status in human cancers remains incompletely understood.

**Methods:**

We developed a next-generation sequencing (NGS)-based pipeline to detect viral sequences from the whole transcriptome and used machine learning algorithms to classify different TIME subtypes.

**Results:**

We revealed a pan-cancer landscape of viral expressions in human cancers where 9 types of viruses were detected in 744 tumors of 25 cancer types. Viral infections showed different tissue tendencies and expression levels. Multi-omics analyses further revealed their distinct impacts on genomic, transcriptomic and immune responses. Epstein-Barr virus (EBV)-infected stomach adenocarcinoma (STAD) and Human Papillomavirus (HPV)-infected head and neck squamous cell carcinoma (HNSC) showed decreased genomic variations, significantly altered gene expressions, and effectively triggered anti-viral immune responses. We identified three TIME subtypes, in which the “Immune-Stimulation” subtype might be the promising candidate for immunotherapy. EBV-infected STAD and HPV-infected HNSC showed a higher frequency of the “Immune-Stimulation” subtype. Finally, we constructed the eVIIS pipeline to simultaneously evaluate viral infection and immune status in external datasets.

**Conclusions:**

Viral infections are prevalent in human cancers and have distinct influences on hosts. EBV and HPV infections combined with the TIME subtype could be promising biomarkers of immunotherapy in STAD and HNSC, respectively. The eVIIS pipeline could be a practical tool to facilitate clinical practice and relevant studies.

**Supplementary Information:**

The online version contains supplementary material available at 10.1186/s12885-021-08871-9.

## Background

Human oncogenic viruses have been implicated in causing 10–15% of human cancers worldwide [1]. They can lead to carcinogenesis directly or indirectly by influencing many of the hallmarks of human cancers, such as sustaining proliferative signaling, triggering genome instability and mutation, eliciting inflammation and avoiding immune destruction [2]. The exploration of virus-cancer associations is critical, as it provides identifiable targets for the prevention, diagnosis and treatment of human cancers [3–6]. Hypothesis-driven methods through epidemiology and low-throughput investigations were the primary methods to study virus-cancer associations. These methods had great limitations in efficiency and have caused false associations [7, 8]. With the advent of next-generation sequencing (NGS), successful efforts have been made in screening viral sequences in a high-throughput fashion. Moreover, large cohorts, such as The Cancer Genome Atlas (TCGA) database, combined with bioinformatics techniques further inspired research of detecting viral sequences in human genomes [9–11].

Immunotherapy has revolutionized the therapeutic strategies of human cancers. The presence of programmed cell death 1 ligand 1 (PD-L1), microsatellite instability-high (MSI-high) or DNA mismatch-repair deficiency (dMMR) and tumor mutation burden (TMB) are the most promising biomarkers for immunotherapy. However, these biomarkers have limited ability in selecting responders [12, 13]. Besides, patients lacking PD-L1, MSI-high or high TMB markers could lose the therapeutic opportunity. Thus finding effective biomarkers in this group of patients is critically urgent. Recently, a study exploring viral infections in six cancers revealed that certain viral infections can shape tumor immune microenvironment (TIME) with altered immune cellular infiltrations. Interestingly, these infected tumors harbor neither high TMB nor MSI-high markers. This indicates viral infections are likely to be an independent biomarker of immunotherapy in certain cancers [14]. Furthermore, several clinical trials have reported a higher overall response to anti-PD treatment in virus-positive cohorts, including squamous cell carcinoma of the head and neck [15], Merkel cell polyomavirus [16, 17], Hodgkin lymphomas and post-transplant lymphoproliferative disorders [18]. And the potential of virus-cancer associations to be applied to immunotherapy has also been reported in several other studies [19, 20]. However, the landscape of viral expressions, immune status and the interplay between them in human cancers remain incompletely understood.

In this study, we aim to investigate viral sequences across human cancers and find their influences on the genome, transcriptome and TIME of their hosts. Also, we aim to generate an integrated pipeline to detect viral infections and identify TIME subtypes. Hopefully, the revealed virus-cancer associations and the developed tools may provide insights into immunotherapy in human cancers.

## Methods

### Datasets and viral sequences detection pipeline

We downloaded 11,206 TCGA BAM format files of 33 cancer types from The Genomic Data Commons (GDC) data portal with official authorization. We aligned raw RNA-seq data in BAM files which came from STAR with reference sequences of human and viral genomes to detect viral expression. Next, we employed StringTie (version 1.3.3) to assemble transcripts for each BAM file, with GENCODE v22 as the reference annotation. Finally, the expression level of each transcript was normalized into TPM (transcripts per kilobase million). For transcripts of the same viral infection type, we selected the maximum TPM value as the final viral mRNA expression. Of note, we refer to an infected case as a tumor infected by a specific virus type, for example, an HPV-infected tumor of HNSC is an HPV infection case in HNSC. By comparison, an infected sample is a sample harboring viral sequences, no matter how many types of viral sequences were detected. Therefore, in samples co-infected by different viruses, the number of infected cases is not equal to that of infected samples or tumor samples.

MAF format files, gene expression profiles (fragments per kilobase million, FPKM) and corresponding clinical information of 11,206 tumor samples were also downloaded from the TCGA database. Information of total leukocyte fraction (LF), 22 types of lymphocyte infiltration, genomic features (including silent mutation rate, nonsilent mutation rate, copy number variation (CNV), aneuploidy score, homologous recombination defects (HRD), intratumor heterogeneity (ITH)) of each tumor sample was obtained from a previous study [21]. All data involved in the analysis is available in Supplementary Table 1.

### TMB calculation

Tumor mutation burden (TMB) is defined as the number of somatic mutations (excluding germline mutations) within the whole genome. In this study, the TMB of each tumor sample was calculated by measuring mutations per megabase (Mb) based on MAF format files from TCGA. For each sample, we merged all somatic mutations calculated by four different techniques, including MuTect [22], MuSE [23], VarScan [24], and SomaticSniper [25]. 40 Mb was considered as the size of the exome, and the final TMB is calculated as the number of somatic mutations divided by 40. According to the previous study [26], tumor samples were further partitioned into 3 groups: samples with 1–5 mutations/Mb are defined as “Low-TMB”; samples with 6–19 mutations/Mb are defined as “Intermediate-TMB”; samples with > = 20 mutations/Mb are defined as “High-TMB”. TMB of each sample is available in Supplementary Table 1.

### Criteria of segregating 22 leukocyte subtypes into 9 subsets

We segregated 22 leukocyte subtypes into 9 subsets according to the criteria from a previous study [21]:

T.cells.CD8 = T.cells.CD8,

T.cells.CD4 = T.cells.CD4.naive+T.cells.CD4.memory.resting+T.cells.CD4.memory.activated+T.Cells.Follicular.Helper+T.Cells.gamma.delta+T.Cells.Regulatory.Tregs,

B.cells = B.cells.naive + B.cells.memory + Plasma.Cells,

NK.cells = NK.cells.resting + NK.cells.activated,

Macrophage = Macrophages.M0 + Macrophages.M1 + Macrophages.M2,

Dendritic.cells = Dendritic.cells.resting + Dendritic.cells.activated,

Mast.cells = Mast.cells.resting + Mast.cells.activated,

Neutrophils = Neutrophils + Monocytes,

Eosinophils = Eosinophils.

The original data of 22 leukocyte subtypes was obtained from Ref (21). The original data and aggregated data are available in Supplementary Table 1.

### Gene set variation analysis

Gene set variation analysis (GSVA) was implemented using the “GSVA” R package (3.8). Sixteen immune-related pathways were obtained from the Molecular Signatures Database (MSigDB) [27, 28], including:

1. KEGG_B_CELL_RECEPTOR_SIGNALING_ PATHWAY
2. KEGG_CELL_ADHESION_MOLECULES_ CAMS
3. KEGG_CHEMOKINE_SIGNALING_PATHWAY
4. KEGG_COMPLEMENT_AND_COAGULATION_CASCADES
5. KEGG_CYTOKINE_CYTOKINE_RECEPTOR_INTERACTION
6. KEGG_FC_EPSILON_ RI_SIGNALING_ PATHWAY
7. KEGG_FC_GAMMA_R_ MEDIATED_ PHAGOCYTOSIS
8. KEGG_LEUKOCYTE_TRANSENDOTHELIAL_MIGRATION
9. KEGG_NATURAL_KILLER_CELL_MEDIATED_CYTOTOXICITY
10. KEGG_NOD_LIKE_RECEPTOR_SIGNALING_PATHWAY
11. KEGG_RIG_I_LIKE_RECEPTOR_SIGNALING_PATHWAY
12. KEGG_T_CELL_RECEPTOR_SIGNALING_PATHWAY
13. KEGG_TGF_BETA_SIGNALING_ PATHWAY
14. KEGG_TOLL_LIKE_RECEPTOR_SIGNALING_PATHWAY
15. REACTOME_PD1_SIGNALING
16. BIOCARTA_CTLA4_PATHWAY

Detailed information about GSVA is available in a previous study [29].

### Differential expression analysis

Gene expressions (FPKM) were log2-transformed after adding one as the pseudo count and then processed by the “limma” R package (3.36.5). Differentially expressed genes (DEGs) were defined as genes with $\left|{\mathit{\log}}_2^{FC}\right|>0.5$ and adjusted *P* < 0.05. Enrichment analyses were performed using the “clusterProfiler” R package [30].

### Soft clustering analysis

Using whole DEGs for enrichment analysis can cause redundancies which is much less biologically interpretable. In this case, subtle changes would be masked by dominant alterations. Therefore, we chose fuzzy c-means (FCM) to divide whole DEGs into different functional modules. FCM is a soft clustering approach and we implemented the method using the “Mfuzz” R package (3.8) [31, 32]. After excluding genes with low standard deviation and standardization, DEGs profiles were converted into “ExpressionSet” objects. Fuzzier “m” was identified using the “mestimate” function and the number of clusters was adjusted by the “Dmin” function where the turning point in the decline curve of the minimum centroid distance was selected. Cluster comparisons were implemented using the “compareCluster” function of “clusterProfiler” R package (3.8.1).

### K-means unsupervised clustering

We performed clustering analysis on the “LF-high” samples of six cancers including LGG, COAD, CESC, KIRC, UVM and SKCM [33]. These cancers were reported covering various immune environment types. We used infiltration levels of 22 immune cell subtypes and expression levels of 76 immune-related genes as features (Supplementary Table 3). Features were first scaled and the K-means clustering algorithm was performed by the “kmeans” function in R (version 4.0.1). The optimal number of the cluster was determined by the “NbClust” function.

### Kaplan-Meier analysis

The Kaplan-Meier analysis was used to estimate the empirical survival probabilities. Differences between survival curves were tested by the log-rank test using the “survival” R package (version 3.2–7).

### TIDE and IFNG score prediction

We first normalized raw FPKM values according to the recommended way (http://tide.dfci.harvard.edu/). First, raw FPKM values were log2 transformed after adding a pseudo value of one (log2(FPKM+ 1)). For each gene, we then subtracted the mean value calculated by averaging normalized FPKM values across all patients of the same cancer type. Finally, we uploaded the normalized tab files of the expression matrix to the website to get TIDE predictions. The results include “Responder”, “TIDE score”, “IFNG score”, “Dysfunction score”, and “Exclusion score”, etc. (Supplementary Table 5).

### Construction of LF.Score

Leukocyte fraction (LF) distributions were first explored in 9692 tumors of 30 cancer types with available LF information (including ACC, BLCA, BRCA, CESC, CHOL, COAD, ESCA, GBM, HNSC, KICH, KIRC, KIRP, LGG, LIHC, LUAD, LUSC, MESO, OV, PAAD, PCPG, PRAD, READ, SARC, SKCM, STAD, TGCT, THCA, UCEC, UCS and UVM). We found LFs of cancers lacking leucocyte infiltrations [33] were mostly below the 25th percentile of LFs (0.086) in all samples). Using this threshold, samples with LF > = 0.086 (the 25th percentile of LF) were assigned to the “LF-high” group and samples with LF < 0.086 were assigned to the “LF-low” group.

To model a LF.Score that classifies tumors into the “LF-high” or the “LF-low” group based on gene expression profiles, we used TCGA datasets for both training and internal validations. Since TCGA datasets are comprised of different tumor types with unbalanced sample sizes, we adopted stratified sampling and randomly assigned samples of each cancer type into a training set and two validation sets at the same ratio of 4:3:3 (3865, 2931 and 2911 samples for training, validation1 and validation2 cohorts). For feature selection, we first performed correlation analyses in the training cohort. We found 167 protein-coding genes were relevant to LF levels (Pearson’s r > 0.5). Then we performed LASSO regression, which is an L1 regularization technique, to further shrink the size of the gene signature. 10-fold cross-validation LASSO regression was performed using the “cv.glmnet” function in the “glmnet” R package to define the optimal lambda (λ = 0.00673) (Supplementary Fig. S8). Thirty genes remained in the final gene signature and their weights were rescaled between 0 and 1 as the final LF.Score:

LF.Score = 0.77*AMICA1 + 0.27*ARHGAP30 + 1*CCR4 + 0.62*CD247 + 0.26*CD5 + 0.26*CD86 + 0*FGD2 + 0.25*FMNL1 + 0.55*FOXP3 + 0.38*FYB + 0.32*GPR65 + 0.24*GPSM3 + 1*GRAP2 + 0.26*HCLS1 + 0.25*HLA.DPB1 + 0.25*HLA.E+ 0.6*KLRB1 + 0.41*LILRB2 + 0.59*LILRB3 + 0.4*NCF2 + 0.33*NLRC5 + 0.38*PTAFR+ 0.28*RASSF5 + 0.28*SELPLG+ 0.41*SLAMF8 + 0.43*SNX20 + 0.25*SRGN+ 0.72*TIGIT+ 0.25*TNFRSF1B + 0.67*ZNF831.

The performance of the LF.Score in separating the “LF-low” group from the “LF-high” group showed an area under curve (AUC) of 0.855 in the training cohort (95% CI: 0.841–0.868) (Supplementary Fig. S7). Validation1 gained an AUC of 0.847 (95% CI: 0.832–0.863) (Supplementary Fig. S7). Validation2 gained an AUC of 0.851 (95% CI: 0.835–0.866) (Supplementary Fig. S7). We selected LF.Score = 81.03 that got the highest youden index as the cutoff.

### Construction of the support vector machine (SVM) classifier

The SVM classifier is built for distinguishing the “Immune-Anergy” subtype from the “Immune-Stimulation” subtype. We used samples of LGG, COAD, CESC, KIRC, UVM and SKCM from TCGA to develop the model. The “Immune-Anergy” and “Immune-Stimulation” subtypes of these samples were first randomly assigned into a training cohort (1200 samples) and a validation cohort (513 samples). For feature selection, DEG analysis was performed in the training cohort where thirty-seven genes were found differentially expressed between two subtypes ($\left|{\mathit{\log}}_2^{FC}\right|>2$ and adjusted *p* < 0.05). An SVM model was then trained using the 10-fold cross-validation strategy by the “e1071” R package. The efficiency of the classifier was evaluated by the receiver operating characteristic curve (ROC) calculated by the“pROC” R package. The classifier could distinguish the “Immune-Stimulation” subtype from the “Immune-Anergy” subtype in both training cohort (AUC = 0.99, 95% CI: 0.997 to 0.999) (Supplementary Fig. S9) and validation cohort (AUC = 0.995, 95% CI: 0.991 to 0.999) (Supplementary Fig. S9). The best cut-off threshold showed great performance in both training (sensitivity: 99%, specificity: 94%, accuracy: 0.98) (Supplementary Fig. S9) and validation (sensitivity: 98%, specificity: 93%, accuracy: 0.97) (Supplementary Fig. S9) cohorts.

### Construction of eVIIS pipeline

Based on the LF.Score classifier and the SVM classifier, we developed the eVIIS pipeline. The pipeline was built using Python (version 3.6.0) and shell language. It takes several steps to predict viral infections and the TIME subtype: (1) align RNA-seq data to human and viral reference genome sequences using STAR (version <= 2.5) [34]; (2) assemble mapped reads by StringTie (version <= 1.2.3) [35] to detect viral sequences; (3) obtain read counts of genes by featureCounts (version > = 1.5.0) [36], quantify expression levels of genes of FPKM values, and predict the immune status (TIME). The expression of a virus type within a tumor is defined as the maximum TPM value of the multiple transcripts (TPM) from the assembly results generated by StringTie. For TIME prediction, eVIIS quantifies gene expression by normalizing counts into FPKM, according to the formulas provided in the GDC mRNA analysis pipeline:

$$ FPKM=\frac{RC_g\ast {10}^9}{RC_{pc}\ast L} $$

where *RC*_*g*_ and *L* are the number of counts mapped to the gene and the total length of exons for the gene, respectively. *RC*_*pc*_ indicates the number of reads mapped to all protein-coding genes (excluding mitochondrial genes). TIME prediction was implemented based on the two models (LASSO regression and SVM) derived from the TCGA training cohort. For a given sample, eVIIS first calculates its LF.Score using the LASSO regression model based on FPKM values of 30 LF-relevant genes. The sample will be classified as the “Immune-Exclusion” subtype if LF.Score < 81.03. Samples with LF.Score > = 81.03 will be further processed by the SVM model. The SVM model evaluates each sample by the 37 DEGs (FPKM) and predict the sample as the “Immune-Stimulation” or the “Immune-Anergy” subtype. The eVIIS pipeline is available at https://github.com/HuangLab-Fudan/eVIIS.

### Statistical analyses

Wilcoxon rank-sum test was used to compare continuous variables. Pearson’s Chi-squared test was used to compare unordered categorical variables. Correlation analysis was performed by Spearman’s rank correlation. All tests were two-tailed with *p* < 0.05 as a significance cutoff. All statistical analyses were performed using R (version 4.0.1).

## Results

### The landscape of viral expressions in human cancers

**Fig. 1:**
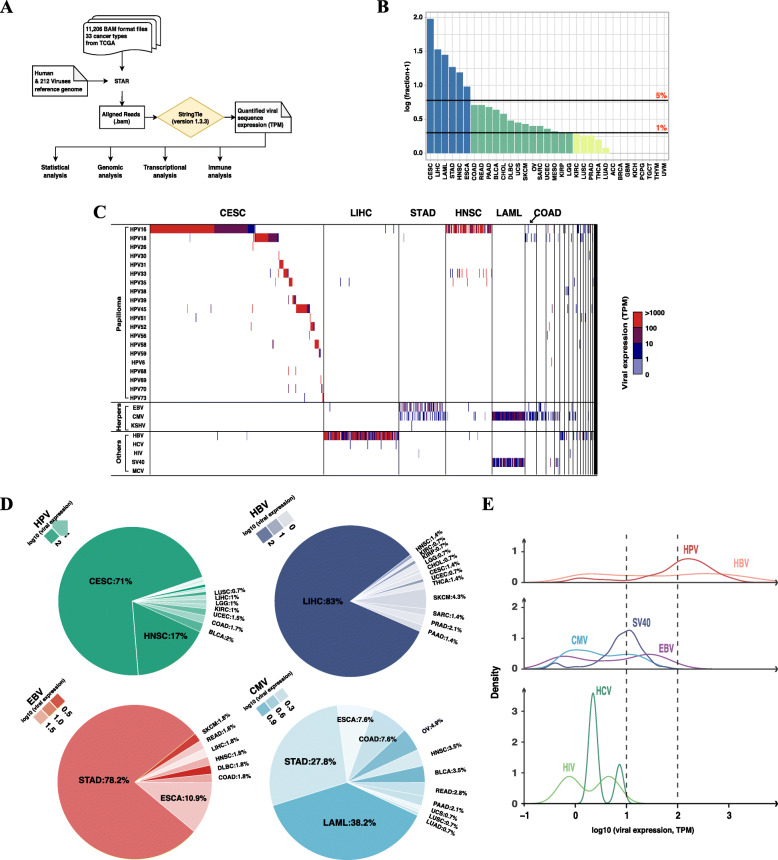
The landscape of viral expressions in human cancers. **A** Pipeline of viral sequence detection and downstream analyses. We downloaded 11,206 BAM files, expression profiles (fragments per kilobase million, FPKM) and clinical data including 33 cancer types from TCGA. For viral sequence detection, we aligned raw RNA-seq data in BAM files came from STAR with reference genomic sequences of human and 212 types of virus to detect viral sequences. We assembled transcripts with StringTie and the expression level of each transcript was normalized by TPM (transcripts per kilobase million). For transcripts of the same viral infection type, the maximum TPM value was selected as the final viral mRNA expression. Viral expression data, RNA-seq expression data and clinical data were then used for downstream statistical, genomic, transcriptional and immune analyses. **B** Fractions of viral infection in human cancers. The value of the fraction of viral infection is log10-transformed after adding a pseudo value of one. 20 types of cancer showed viral infections in over 1% of tumors, and 6 types of cancers showed viral infections in over 5% of tumors. **C** The landscape of viral expressions in human cancers. We showed viral expressions of all 744 infected samples from 25 types of cancer. Cancers are ordered according to the amount of infected samples. Cancers with highest amounts of viral infections are labelled, including CESC, LIHC, STAD, HNSC, LAML and COAD. The unlabeled cancers are ESCA, BLCA, OV, SKCM, READ, PAAD, UCEC, LGG, KIRC, SARC, PRAD, LUSC, KIRP, THCA, CHOL, DLBC, UCS, MESO, LUAD (from left to right). We detected 9 types of viral sequences in total, with 20 subtypes in HPV infections. Viral expressions were normalized into TPM values which are indicated with discrete colors notations. While most samples have a dominant type of viral infection, co-infections are also widespread. **D** Distributions of HPV, HBV, EBV and CMV infections in human cancers. Some cancers with low fractions are not labeled. The color of each sector indicates the average viral expression of tumors of that cancer type. **E** Distributions of viral expression levels. We plotted Kernel density plot for each virus (except for KSHV and MCV which only have a single sample). Viral expressions were log10-transformed and viruses are grouped based on their average expression levels

We designed a pipeline to detect 212 types of viral sequences by interrogating RNA-Seq data of 11,206 tumor samples of 33 cancer types from TCGA (Fig. 1A). Overall, 9 types of viral sequences were detected in 744 tumors of 25 cancer types (Table 1). Over 1% of tumors were found infected in 20 cancer types and over 5% of tumors were infected in 6 cancer types (Fig. 1B). The well-known oncogenic virus-related cervical squamous cell carcinoma and endocervical adenocarcinoma (CESC, 94.14%) and liver hepatocellular carcinoma (LIHC, 33.16%) exhibited the highest proportions of viral infections, followed by acute myeloid leukemia (LAML, 27.50%), stomach adenocarcinoma (STAD, 17.61%), head and neck squamous cell carcinoma (HNSC, 14.58%) and esophageal carcinoma (ESCA, 8.65%). A comprehensive landscape of viral expressions of all infected tumors is presented in Fig. 1C.

**Table 1:** Viral infection in 33 cancer types from TCGA

TCGA	Tumor samples	Infected samples	Infected fraction	Infected cases								
				HPV	HBV	EBV	CMV	HCV	HIV	KSHV	MCV	SV40
**CESC**	307	289	94.10%	311	2	0	0	0	0	0	0	0
**LIHC**	377	125	33.20%	4	117	1	0	4	0	0	0	0
**LAML**	200	55	27.50%	0	0	0	55	0	0	0	0	46
**STAD**	443	78	17.60%	1	0	43	40	0	1	1	0	0
**HNSC**	528	77	14.60%	69	2	1	5	0	0	0	0	0
**ESCA**	185	16	8.60%	0	0	6	11	0	0	0	0	0
**COAD**	459	19	4.10%	7	0	1	11	0	0	0	0	0
**READ**	169	7	4.10%	1	0	1	4	0	1	0	0	0
**PAAD**	185	7	3.80%	2	2	0	3	0	0	0	0	0
**BLCA**	412	14	3.40%	8	0	0	5	0	0	0	0	1
**CHOL**	36	1	2.80%	0	1	0	0	0	0	0	0	0
**DLBC**	50	1	2.00%	0	0	1	0	0	0	0	0	0
**UCS**	57	1	1.80%	0	0	0	1	0	0	0	0	0
**SKCM**	470	8	1.70%	1	6	1	0	0	0	0	0	0
**OV**	602	9	1.50%	1	0	0	7	0	0	0	1	5
**SARC**	261	4	1.50%	2	2	0	0	0	0	0	0	0
**UCEC**	559	7	1.30%	6	1	0	0	0	0	0	0	0
**MESO**	87	1	1.10%	1	0	0	0	0	0	0	0	0
**KIRP**	291	3	1.00%	2	1	0	0	0	0	0	0	0
**LGG**	516	5	1.00%	4	1	0	0	0	0	0	0	0
**KIRC**	535	5	0.90%	4	1	0	0	0	0	0	0	0
**LUSC**	504	4	0.80%	3	0	0	1	0	0	0	0	0
**PRAD**	498	4	0.80%	1	3	0	0	0	0	0	0	0
**THCA**	507	3	0.60%	1	2	0	0	0	0	0	0	0
**LUAD**	580	1	0.20%	0	0	0	1	0	0	0	0	0
**ACC**	92	0	0.00%	0	0	0	0	0	0	0	0	0
**BRCA**	1098	0	0.00%	0	0	0	0	0	0	0	0	0
**GBM**	599	0	0.00%	0	0	0	0	0	0	0	0	0
**KICH**	66	0	0.00%	0	0	0	0	0	0	0	0	0
**PCPG**	179	0	0.00%	0	0	0	0	0	0	0	0	0
**TGCT**	150	0	0.00%	0	0	0	0	0	0	0	0	0
**THYM**	124	0	0.00%	0	0	0	0	0	0	0	0	0
**UVM**	80	0	0.00%	0	0	0	0	0	0	0	0	0
**SUM**	11,206	744		429	141	55	144	4	2	1	1	52

For CESC, all infected tumors were related to Human Papillomavirus (HPV), in which the high-risk HPV16 and HPV18 dominantly accounted for 60.90 and 14.53%, respectively. Besides, we detected 2 cases of HBV infection in CESC. For LIHC, 93.6% tumors (117 out of 125) were infected by Hepatitis B virus (HBV). Additionally, four tumors were infected by HPV (HPV16 and HPV35), four tumors were infected by Hepatitis C virus (HCV), and one tumor was infected by Epstein-Barr virus (EBV). For STAD, 55.1% of tumors (43 out of 78) were infected by EBV and 51.3% of tumors (40 out of 78) were infected by Cytomegalovirus (CMV). Besides, one tumor was infected by HPV18, one tumor was infected by Kaposi’s sarcoma-associated herpesvirus (KSHV), and one tumor was infected by Human Immunodeficiency Virus (HIV). Of note, we found co-infection of EBV and CMV occurred frequently in gastrointestinal tumors. Apart from STAD, ESCA showed 3.2% of tumors (6 out of 185) with EBV infection and 5.9% of tumors (11 out of 185) with CMV infection. For HNSC, 89.61% of tumors (69 out of 77) were infected by HPV and HPV16 was the dominant subtype (79.7%; 55 out of 69). Also, we found one tumor infected by EBV, 3 tumors infected by CMV, 2 tumors infected by HBV. For LAML, we detected CMV infection in all tumors and SV40 infection in 83.6% of tumors (46 out of 55). We compared the above results with the clinical information provided by TCGA. For HPV infection, 22 out of 23 records of CESC and 91 out of 96 records of HNSC agreed with our detection. Additionally, we detected other subtypes of HPV, including HPV45, HPV52, HPV58 and HPV70. As for EBV infection, EBV infections were detected in 27 out of 30 tumors that were accordingly defined as the GI.EBV subtype by TCGA (Supplementary Table 1) [37].

Within the nine types of viruses we detected, HPV, HBV, EBV and CMV are the most prevalent types. We found these viral infections showed different tissue tendencies (Fig. 1D). HPV infections occurred mostly in CESC (71%) and HNSC (17%); HBV infections occurred mostly in LIHC (83%); EBV infections occurred mostly in STAD (78.2%) and ESCA (10.9%); and CMV infections occurred mostly in LAML (38.2%), STAD (27.8%), colon adenocarcinoma (COAD; 7.6%) and ESCA (7.6%). Besides their main hosts, these viruses were sporadically detected in a wide range of human cancers. For example, HPV infections were also detected in bladder urothelial carcinoma (BLCA; 2%), COAD (1.7%) and uterine corpus endometrial carcinoma (UCEC; 1.5%); HBV infections were also detected in skin cutaneous melanoma (SKCM; 4.3%), prostate adenocarcinoma (PRAD; 2.1%); CMV infections in ovarian serous cystadenocarcinoma (OV; 4.9%), HNSC (3.5%), BLCA (3.5%), rectum adenocarcinoma (READ; 2.8%) and pancreatic adenocarcinoma (PAAD; 2.1%). Compared to the main hosts, viral expressions were generally lower in these uncommon hosts. However, some uncommon hosts exhibited high viral expressions, such as HPV-infected BLCA, HPV-infected UCEC, HBV-infected HNSC, HBV-infected KIRP, HBV-infected UCEC, EBV-infected SKCM, EBV-infected DLBC, CMV-infected OV and CMV-infected BLCA (Fig. 1D). As for the less prevalent types, 46 tumors of LAML and 5 tumors of OV were infected by Simian virus 40 (SV40); 4 tumors of LIHC were infected by Hepatitis C virus (HCV); 1 tumor of STAD and 1 tumor of READ were infected by Human Immunodeficiency Virus (HIV); 1 tumor of STAD was infected by Kaposi’s sarcoma-associated herpesvirus (KSHV); and 1 tumor of OV was infected by Merkel cell polyomavirus (MCV) (Table1). These nine viruses showed considerably varied viral expressions. HPV and HBV were the highest, followed by CMV, EBV and SV40, and HCV and HIV showed the lowest levels of viral expressions (Fig. 1E; Table 2).

**Table 2:** Reference intervals of viral expressions

	Mean	Median	P_25_-P_75_
HBV	497.79	42.74	2.95–377.14
HPV	234.90	132.45	48.50–280.21
EBV	16.83	4.92	0.65–28.12
SV40	11.14	9.34	6.04–13.84
CMV	7.05	2.47	0.98–9.70
HCV	3.50	2.32	2.21–3.61
HIV	2.65	2.65	1.70–3.59
MCV	0.52		
KSHV	1.14		

**Fig. 2:**
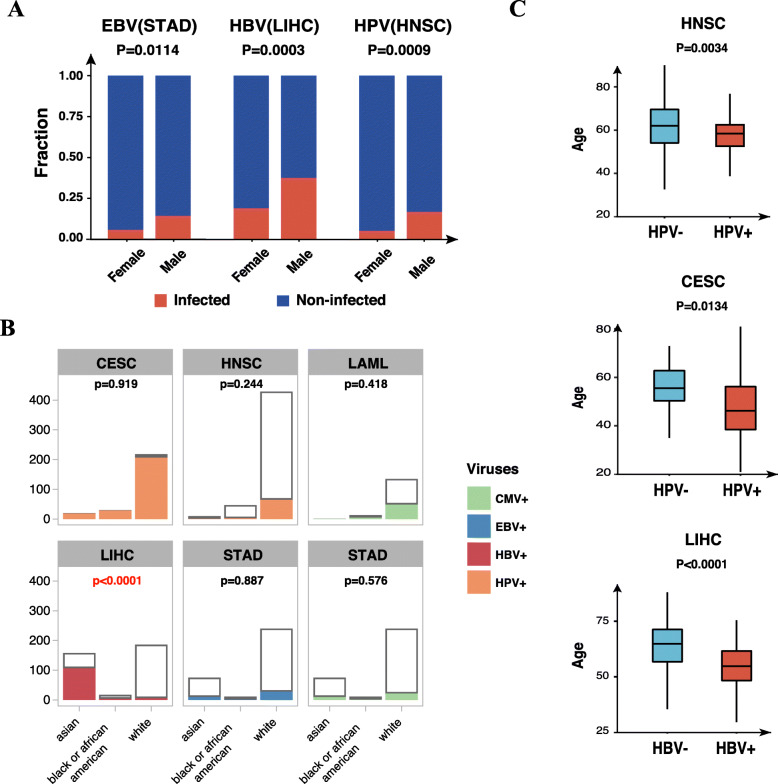
Demographic characteristics of viral infections. **A** Gender characteristic of virus-associated cancers. EBV-, HBV- and HPV-infections appear more frequently in males of STAD, LIHC and HNSC, respectively (*P* < 0.05, Pearson’s Chi-squared test). **B** Germline analysis for viral infections. We analyzed correlations between viral infections and different races in cancers with high proportion of viral infection. HBV infection in LIHC is the only virus-cancer association that has significant difference in germline distribution where LIHC of Asian ancestry is more susceptible to HBV-infection (*P* < 0.0001, Pearson’s Chi-squared test). **C** Age analysis for virus-associated cancers. The age of diagnosis showed earlier in HPV-infected CESC (*P* = 0.0134, Pearson’s Chi-squared test), HPV-infected HNSC (*P* = 0.0034, Pearson’s Chi-squared test), and HBV-infected LIHC (*P* < 0.0001, Pearson’s Chi-squared test) showed an earlier age of diagnosis

We also analyzed demographic characteristics of different viral infections. EBV, HBV and HPV infections appeared more frequently in males of STAD, LIHC and HNSC, respectively (*P* < 0.05, Pearson’s Chi-squared test) (Fig. 2A, Supplementary Table 4). Ancestry analysis showed that, within the most prevalent virus-cancer associations (including EBV-STAD, CMV-STAD, HBV-LIHC, HPV-CESC, HPV-HNSC and CMV-LAML), only HBV infection showed a significant difference in LIHC where tumors of the Asian ancestry were more prone to HBV infection (*P* < 0.0001, Pearson’s Chi-squared test) (Fig. 2B, Supplementary Table 4). HPV-infected CESC (*P* = 0.0134), HPV-infected HNSC (*P* = 0.0034), and HBV-infected LIHC (*P* < 0.0001) showed an earlier age of diagnosis (Pearson’s Chi-squared test) (Fig. 2C, Supplementary Table 4).

### EBV and HPV infections showed decreased genomic variations

**Fig. 3:**
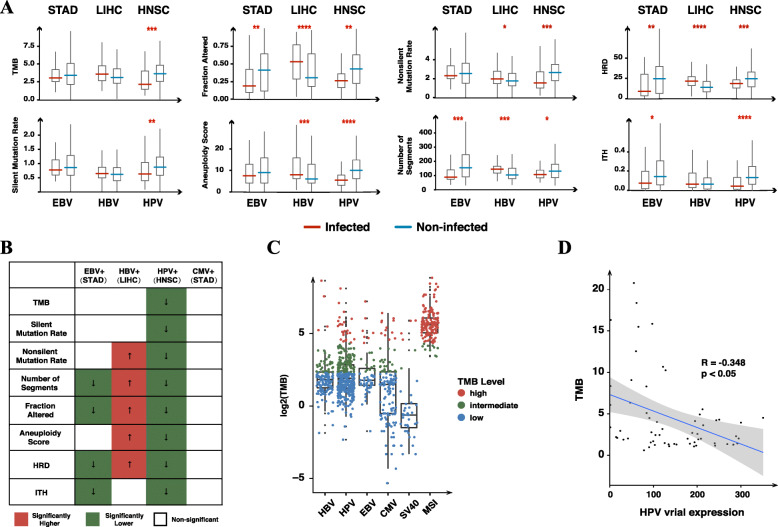
EBV and HPV infections showed decreased genomic variations. **A** Genomic features of EBV-infection, HBV-infection and HPV-infection in STAD, LIHC and HNSC, respectively. Comparisons were made between infected and non-infected tumor samples for each cancer type. Significant results are labeled (*:0.05–0.01; **:0.01–0.001; ***:0.001–0.0001; ****: < 0.0001; two-tailed Mann-Whitney U test). **B** Genomic features of viral infections. We analyzed EBV-infected STAD, CMV-infected STAD, HBV-infected LIHC and HPV-infected HNSC. All genomic features are significantly decreased in HPV-infected HNSC, four features are significantly decreased in EBV-infected STAD. By comparison, five features are significantly increased in HBV-infected LIHC. No significant change were found in CMV-infected STAD (*p* < 0.05; two-tailed Mann-Whitney U test). **C** Comparison between tumors with different viral infections and MSI (microsatellite instability)-high tumors. The red, green and blue dots represent for TMB-high, TMB-intermediate and TMB-low, respectively. **D** Correlation between TMB burden and HPV-infection. TMB levels showed significant negative correlation with RNA expressions in all HPV-infected tumors (*R* = -0.348, *p* < 0.05, Spearman’s rank correlation)

To assess the effects of viral infections upon the host genome, we analyzed tumor mutation burden (TMB), mutation rate (silent and non-silent), copy number variation (CNV, both segment and fraction of genomic alterations), aneuploidy, homologous recombination defects (HRD) and intratumor heterogeneity (ITH) for the most prevalent virus-cancer associations (including EBV infection in STAD, HBV infection in LIHC and HPV infection in HNSC). We found all eight features decreased consistently in HPV-infected HNSC and features including CNV, HRD and ITH were decreased in EBV-infected STAD (*p* < 0.05; two-tailed Mann-Whitney U test). By comparison, five features (SNV, CNV- segment, CNV-fraction, aneuploidy and HRD) were increased in HBV-infected LIHC (*p* < 0.05; two-tailed Mann-Whitney U test) (Fig. 3A and Fig. 3B; Supplementary Table 2). Using TMB as an indicator, we further compared infected tumors with tumors with high microsatellite instability (MSI-high) [38, 39]. Most virus-infected tumors were classified as “intermediate TMB” or “low TMB”, compared to that most MSI-high tumors were classified as “high TMB” (Fig. 3C). Beside, we found a significant negative correlation between HPV expressions and TMB in all HPV-infected tumors (*P* < 0.05, Spearman’s rank correlation) (Fig. 3D).

### EBV and HPV infections displayed significantly changed gene expressions

**Fig. 4:**
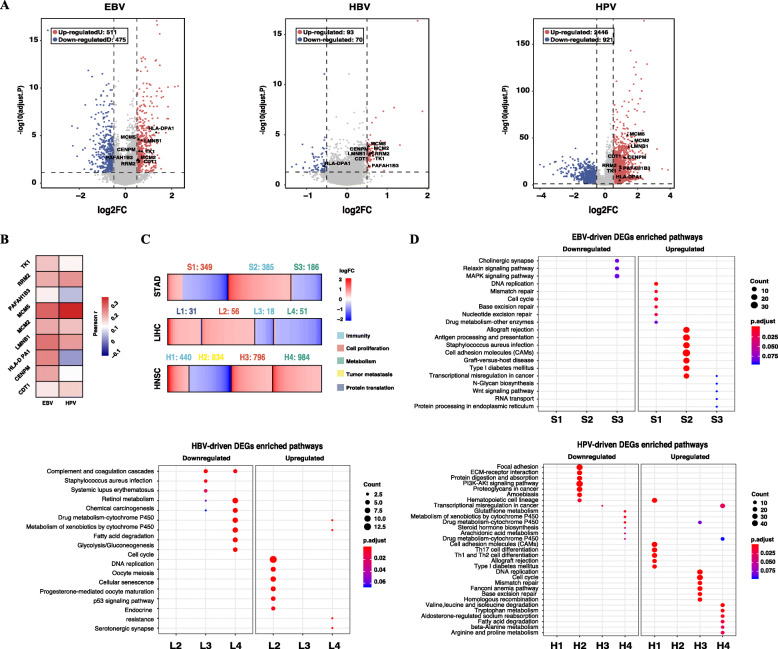
EBV and HPV infections displayed significantly changed gene expressions. **A** Volcano plots of differentially expressed genes (DEGs) regarding EBV-infection in STAD, HBV-infection in LIHC and HPV-infection in HNSC. DEGs are defined as genes with the absolute value of log2-fold change (|log_2_FC|) > 0.5 and adjust.*P* < 0.05. The red dots represent upregulated DEGs and the blue dots represent downregulated DEGs. The total number and |log_2_FC| values of DEGs regarding EBV- and HPV-infection are significantly larger than DEGs regarding HBV-infection. Nine genes are overlapped in all types of viral infection-regarding DEGs which are annotated in the figure. (**B**) Correlations between mRNA expressions of the nine overlapped DEGs and EBV, HPV viral expressions in STAD and HNSC, respectively (Pearson’s correlation). **C** Heatmap of viral infection-regarding DEGs in each gene subset for STAD, LIHC and HNSC. The color bar indicates the scale of the log_2_FC value for each DEG. The total number of genes in each gene subset is annotated above and the corresponding biological function are labeled with different colors. **D** Pathway analysis for different viral infection-regrading DEGs. Comparisons were made of enriched pathways of DEGs in different gene subsets for each cluster. Upregulated and downregulated DEGs were analyzed separately. The number of DEGs enriched in a pathway is indicated by the size of the circle and the color indicates the corresponding adjust.*P* value

To explore the impact of viral infections upon the transcriptome, we performed differential expression analysis. For HPV-infected HNSC, we identified 3367 differentially expressed genes (DEGs), of which 2446 DEGs were upregulated and 921 DEGs were downregulated. For EBV-infected STAD, we identified 986 DEGs, of which 511 DEGs were upregulated and 475 DEGs were downregulated. In contrast, only 163 DEGs were identified in HBV-infected LIHC, of which 93 DEGs were upregulated and 70 DEGs were downregulated. Compared to HPV-related DEGs and EBV-related DEGs, changes of HBV-related were much smaller. For all virus-related DEGs, 9 genes were overlapped, including CDT1, CENPM, HLA-DPA1, LMNB1, MCM2, MCM5, PAFAH1B3, RRM2 and TK1 (Fig. 4A). Expressions of these genes were positively correlated with the expressions of EBV and HPV (Fig. 4B). Except that HLA-DPA1 is related to antigen presentation, the rest genes are exclusively associated with cell proliferation. To gain a better understanding of the biological functions of these DEGs, we performed fuzzy c-means (FCM) clustering analysis. We identified 3 gene clusters in STAD (S1, S2, S3), 4 gene clusters in LIHC (L1, L2, L3, L4) and 4 gene clusters in HNSC (H1, H2, H3, H4) (Supplementary Fig. 1). 385 DEGs of S2 and 440 DEGs of H1 were exclusively upregulated, while 18 DEGs of L3 were all downregulated. Enrichment analysis showed these gene clusters were mainly associated with immunity. All DEGs in L2 and H3 were upregulated, while a large portion of DEGs of S1 was decreased. Enrichment analysis showed these gene clusters were relevant to cell proliferation (Fig. 4C). Moreover, we found pathways involving DNA replication, mismatch repair, base excision repair, and nucleotide excision repair were upregulated in S1 and H3 (Fig. 4D; Supplementary Fig. S2).

### EBV and HPV infections showed effective anti-viral immune responses and upregulated PD1 signaling pathway

**Fig. 5:**
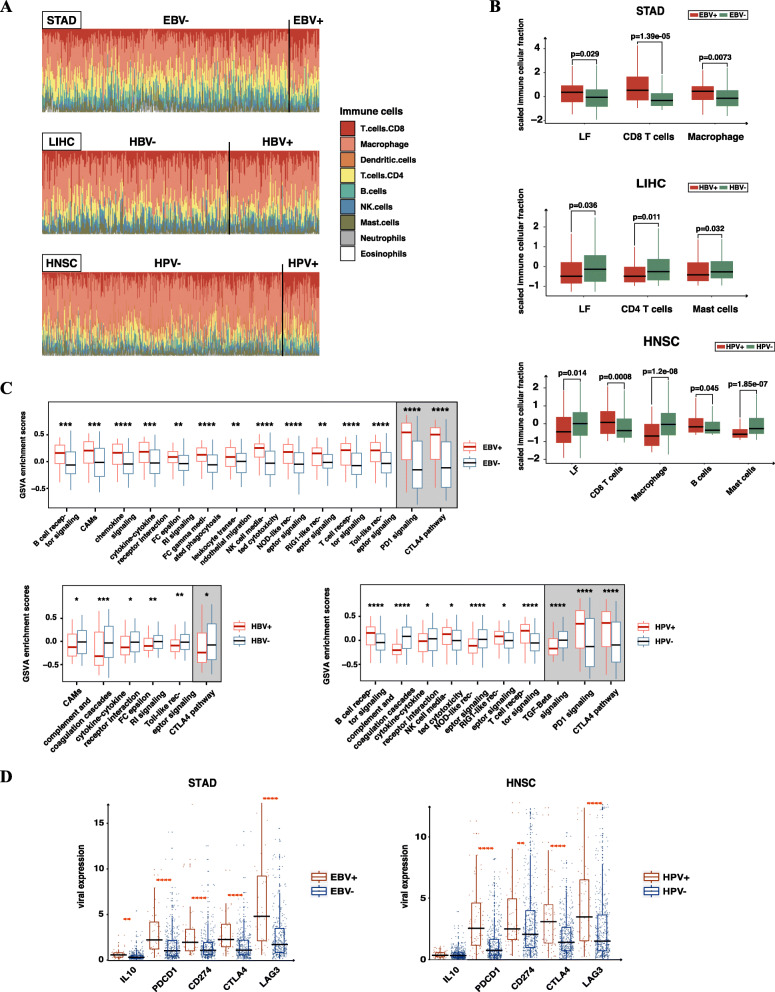
EBV- and HPV-infected tumors showed effective anti-viral immune responses and upregulated PD1 signaling pathway. **A** Landscapes of immune infiltrations in tumors of STAD, LIHC and HNSC. Immune cellular compositions were scaled for each tumor. Non-infected tumors are showed on the left side and infected tumors are showed on the right side. **B** Immune infiltration analysis for different types of viral infections. We compared leukocyte fraction (LF) and nine aggregated immune infiltrations of EBV-, HBV- and HPV-infected tumors and non-infected tumors in STAD, LIHC and HNSC. The figure only shows results with significant difference (*p* < 0.05; two-tailed Mann-Whitney U test). **C** Gene set variation analysis (GSVA) of immune-related gene sets for different viral infections. Expression levels of each immune-related pathway are compared between EBV-infected, HBV-infected and HPV-infected tumors and non-infected tumors of STAD, LIHC and HNSC, respectively. The figure only showed results with significant difference (*:0.05–0.01; **:0.01–0.001; ***:0.001–0.0001; ****: < 0.0001; two-tailed Mann-Whitney U test). Pathways regrading immune activation are shown on the left side and pathways regrading immune suppression are shown on the right side. **D** Immune-suppressive status analysis for EBV- and HPV-infection. Expression levels of five immunosuppressive molecules were compared between EBV-infected and HPV-infected tumors and non-infected tumors for STAD and HNSC (*:0.05–0.01; **:0.01–0.001; ***:0.001–0.0001; ****: < 0.0001; two-tailed Mann-Whitney U test)

We then focused on the influences of viral infections on immune cellular infiltrations. Distributions of the integrated 9 immune cells of STAD, LIHC and HNSC are presented in Fig. 5A. For STAD, the leukocyte fraction (LF) and infiltrations of CD8+ T cells and macrophages were higher in EBV-infected tumors (*p* < 0.05; two-tailed Mann-Whitney U test). For LIHC, LF, CD4+ T cells and mast cells were decreased in HBV-infected tumors (*p* < 0.05; two-tailed Mann-Whitney U test). For HNSC, infiltrations of CD8+ T cells and B cells were increased, while LF, macrophages and mast cells were decreased in HPV-infected tumors (*p* < 0.05; two-tailed Mann-Whitney U test) (Fig. 5B). In all EBV- and HPV-associated cancers, infiltrations of CD8+ T cells were higher in infected tumors (Supplementary Fig. S3). For all EBV-infected tumors, a positive correlation was observed between EBV expressions and infiltration levels of CD8+ T cells (*r* = 0.37, *P* = 0.0086, Spearman’s rank correlation) (Supplementary Fig. S4). Shannon entropy and species richness are indicators of T cell infiltration levels, and TCR evenness could stand for the diversity of T cell receptors. We noticed that Shannon entropy and species richness were increased, while TCR evenness was decreased in EBV-infected STAD. This implies that EBV-infected tumors may have undergone a clonal expansion [14] (Supplementary Fig. S5). This is consistent with the observation that ITH was decreased in EBV-infected tumors.

We further examined how immune-related functions were regulated in these virus-infected tumors [27, 28]. In terms of immune stimulation,12 pathways responsible for immune cell migration and infiltration, antigen recognition, innate immune response and adaptive immune response were upregulated in EBV-infected STAD; and 4 pathways representing B cell receptor signaling, NK cell-mediated cytotoxicity, RIG-1 like receptor signaling and T cell receptor signaling were upregulated in HPV-infected HNSC. As for immune suppression, suppressive pathways like PD1 signaling and CTLA-4 pathways were upregulated in EBV-infected STAD and HPV-infected HNSC (two-tailed Mann-Whitney U test) (Fig. 5C). Immune-suppressive molecules, including LAG3, PDCD1, CD274 (PD-L1), CTLA4 and IL10 were also upregulated in EBV-infected STAD and HPV-infected HNSC (two-tailed Mann-Whitney U test) (Fig. 5D). By comparison, all immune-related pathways were decreased in HBV-infected LIHC (two-tailed Mann-Whitney U test) (Fig. 5C).

### Identification of TIME subtypes

**Fig. 6:**
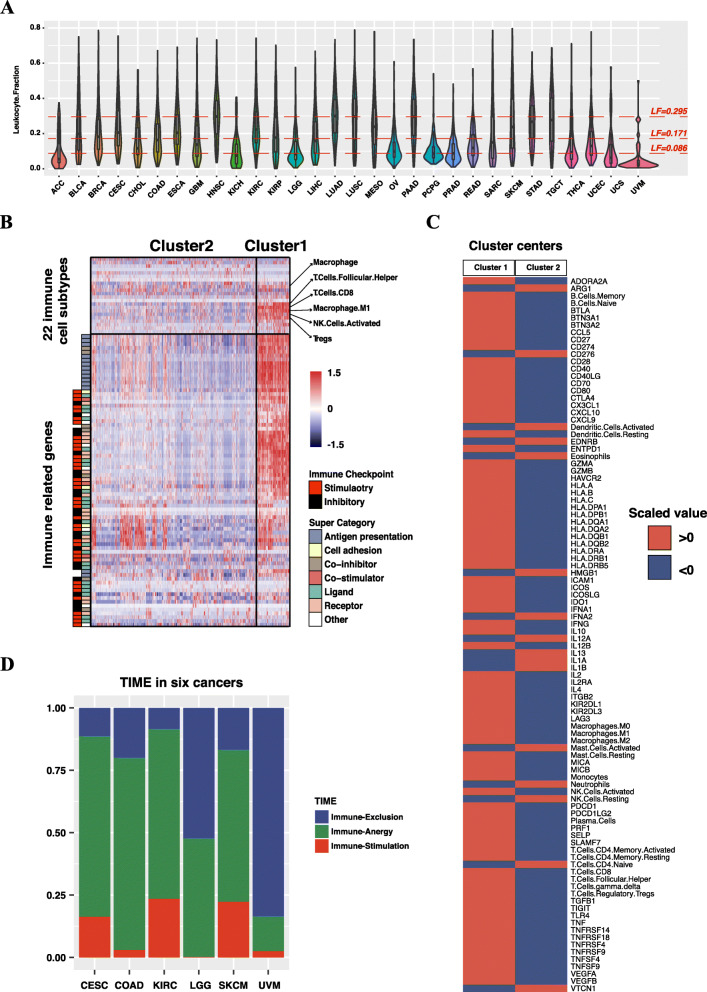
Identification of TIME subtypes. **A** Distributions of leukocyte fractions (LFs) of human cancers. We analyzed LFs of 9692 tumors of 30 different cancer types, and 25th (LF = 0.086), 50th (LF = 0.171) and 75th (LF = 0.295) percentiles of LFs of these tumors are annotated in the figure. (**B**) Heatmap reveals two different clusters in the LF-high tumors. The clustering analysis was implemented using expression levels of 76 immune-related genes and infiltration levels of 22 types of immune cells. All values were scaled before clustering. Important immune cell types are labeled with arrows, and immune checkpoint and super categories of each gene are labeled on the left side. **C** Heatmap of scaled cluster centers of Cluster 1 and Cluster2. Values ​​greater than 0 are in red, values ​​less than 0 are in blue. Labels of 76 immune-related genes and 22 types of immune cells are annotated on the right side. Nearly all expression levels of the immune-related genes and immune cellular infiltrations are higher in Cluster1 than Cluster2. **D** TIME subtypes in LGG, COAD, CESC, KIRC, UVM and SKCM. Based on the LF levels and clustering results, we manually classified training samples of LGG, COAD, CESC, KIRC, UVM and SKCM into three different TIME subtypes, namely “Immune-Stimulation”, “Immune-Anergy” and “Immune-Exclusion” subtypes

Tumor immune microenvironment (TIME) is a prerequisite of applying immunotherapy in the clinic [40]. In this part, we proposed a method to classify tumors into different types of TIME. The general idea of identifying different TIME subtypes is to first separate tumors with high leucocyte infiltrations from those lacking leucocyte infiltrations. For this purpose, we first studied LF distributions of the 30 cancer types with available data from TCGA. We found LFs of cancers lacking leucocyte infiltrations [33] were mostly below the 25th percentile of LFs (0.086) (Fig. 6A). Using this threshold, we separated tumors into a “LF-high” group and a “LF-low” group. Then within the “LF-high” group, we wanted to identify tumors with activated immune responses and those with functionally impaired immune responses. Specifically, we used infiltrations of all 22 types of immune cells and expressions of 76 immune-related genes (Supplementary Table 3) as features to perform an unsupervised clustering analysis on all “LF-high” tumors of LGG, COAD, CESC, KIRC, UVM and SKCM TCGA datasets. We chose these cancer types because they have been reported covering different immune environment subtypes [33]. The clustering analysis identified two different clusters (Fig. 6B). Cluster1 is characterized by increased infiltrations of both cytolytic cells (CD8+ T cells, M1 macrophages, activated NK cells and follicular helper T cells) and immune-suppressive cells (Tregs and M2 macrophages). Accordingly, expressions of cytolytic genes (GZMA, GZMB, PRF1, IFNG and TNF) and immunosuppressive genes (PDCD1, CD274, LAG3 and CTLA4) are also upregulated. By contrast, infiltration levels of immune cells and expressions of immune-related genes are nearly all lower in Cluster2 than Cluster1 (Fig. 6C). LF levels reflect the overall level of immune cellular infiltrations, and different patterns of expressions of 76 immune-related genes and infiltrations of 22 immune cells indicate different functional conditions. By aggregating LF levels and the clustering results, we manually identified three different TIME subtypes. The “LF-low” group is defined as the “Immune-Exclusion” subtype, and Cluster1 and Cluster2 within the “LF-high” group are defined as the “Immune-Stimulation” subtype and the “Immune-Anergy” subtype separately.

We compared our TIME subtyping results with a previous research that identified different immune types across human cancers [41]. LGG and UVM showed higher fractions of the “Immune-Exclusion” subtype and lower fractions of the “Immune-Stimulation” subtype. This result is compatible with the decreased immune responses in LGG and UVM reported by the study. By comparison, CESC, KIRC and SKCM showed relatively higher fractions of the “Immune-Stimulation” subtype, and these cancer types also showed stimulated immune responses in the study(Fig. 6D). TIDE is a tool to predict immune responses for an individual cancer type. It currently only supports predicting for SKCM and non-small cell lung cancer (NSCLC) and the prediction of SKCM is more robust [42]. Therefore, we chose the SKCM TCGA dataset for comparison. Both TIDE and TIME methods evaluate CTL infiltration levels and dysfunction status, and TIME additionally evaluates a stimulated status. For the evaluation of CTL infiltrations, all “CTL-high” tumors are constituted of the “TIME-Stimulation” (62%) and the “TIME-Anergy” (38%) TIME subtypes, and most of “CTL-low” tumors are constituted of the “TIME-Exclusion” (25%) and the “TIME-Anergy” (73%) TIME subtypes (Supplementary Fig. S10). Besides, “T cell exclusion scores” showed a significant negative correlation with “LF scores” (cor = − 0.66; *p*-value < 2.2e-16, Pearson’s correlation) (Supplementary Fig. S10). These results are expected for both “T cell exclusion scores” and “LF scores” are measurements of CTL infiltrations. In addition, we found “T cell exclusion scores” is significantly higher in the “Immune-Exclusion” subtype than the “Immune-Anergy” subtype (mean: 0.88 and 0.35; *p*-value = 5.971e-06, t-test) and the “Immune-Stimulation” subtype (mean: 0.88 and − 1.67; *p*-value < 2.2e-16, t-test); and the “Immune-Anergy” subtype is higher than the “Immune-Stimulation” subtype (mean: 0.35 and − 1.67; *p*-value < 2.2e-16, t-test) (Supplementary Fig. S10). For the measurement of dysfunction status, we found “T cell dysfunction scores” are significantly higher in the “Immune-Stimulation” subtype than the “Immune-Anergy” subtype (mean: 1.16 and − 0.23; *p* < 2.2e-16, t-test) and the “Immune-Exclusion” subtype (mean: 1.16 and − 0.99; *p* < 2.2e-16, t-test); and the “Immune-Anergy” subtype is higher than the “Immune-Exclusion” subtype (mean: − 0.99 and − 0.23; *p* = 2.3e-11, t-test) (Supplementary Fig. S10). These results are expected because, in the “Immune-Stimulation” subtype, cytolytic genes and immunosuppressive genes are co-expressed and upregulated. The “Immune-Stimulation” subtype gets higher “T cell dysfunction scores” for their high expressions of immune-suppressive genes. By comparison, the “Immune-Anergy” subtype lacks immune signals, so “T cell dysfunction scores” are expected to be lower. We also compared expression levels of the IFN-Y signature among TIME subtypes. The “Immune-Stimulation” subtype showed the highest level of “IFNG scores” (mean: 2.09), which is higher than the “Immune-Anergy” subtype (mean: − 0.26; *p* < 2e-16, t-test) and the “Immune-Exclusion” subtype (mean: − 1.68; *p* < 2e-16, t-test). and “IFNG scores” are higher in the “Immune-Anergy” subtype also shows a higher level of “IFNG scores” than the “Immune-Exclusion” subtype (*p* < 2e-16, t-test) (Supplementary Fig. S11). These results are expected as the IFN-Y signal is strengthened by stimulated immune responses and increased immune infiltration.

### Correlations between viral infections and TIME subtypes

To predict TIME subtypes in STAD, LIHC, HNSC, as well as other cancers, we developed a workflow, including a LASSO regression model to predict the LF level and an SVM classifier to separate two clusters. The LF model was trained on the training cohort (3865 samples) and validated in the two validation datasets (2931 and 2911 samples) from the whole TCGA datasets. And the SVM model was trained (1200 samples) and validated (513 samples) on samples of the “Immune-Anergy” and the “Immune-Stimulation” TIME subtypes of LGG, COAD, CESC, KIRC, UVM and SKCM from TCGA datasets (Supplementary Figs. S7, S8, and S9).

**Fig. 7:**
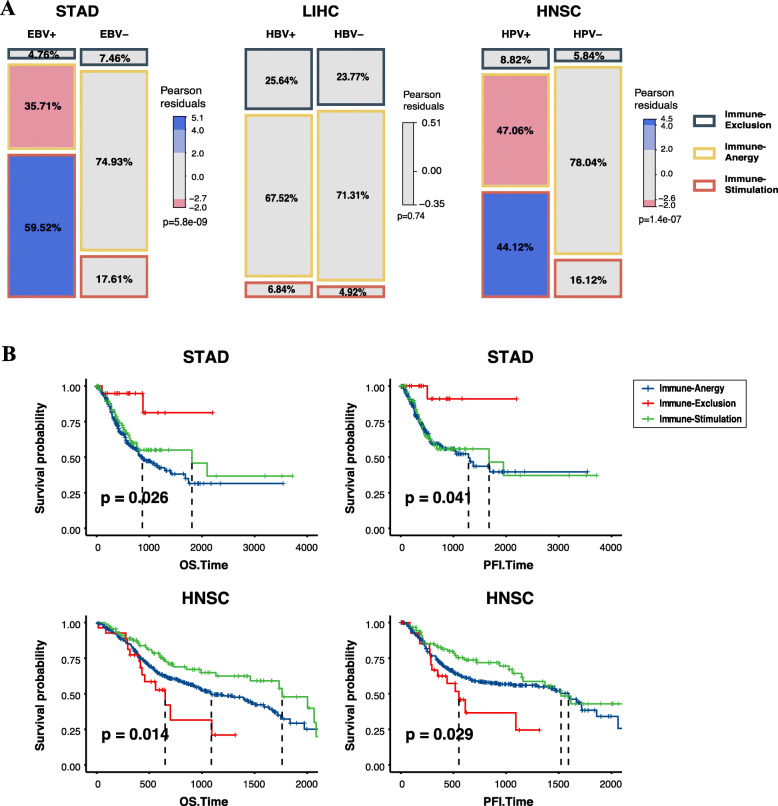
Correlations between viral infections and TIME subtypes. **A** Associations between viral infections and TIME subtypes. We predicted TIME subtypes for tumors of STAD, LIHC and HSNC. We found significant correlations between TIME subtypes and viral infections in STAD and HNSC. By comparison, LIHC showed no significant correlation with TIME subtypes. **B** Survival analyses of different TIME subtypes of STAD and HNSC. The survival curves show better outcomes of overall survival (OS) and progression-free interval (PFI) in the “Immune-Exclusion” subtype of STAD and the “Immune-Stimulation” subtype of HNSC

EBV and HPV significantly influenced the TIME subtypes of STAD (*p* = 5.8e-9, Pearson’s Chi-squared test) and HNSC (*p* = 1.4e-7, Pearson’s Chi-squared test), respectively. However, HBV infection showed no significant impact on the TIME subtypes of LIHC (Fig. 7A). Compared to non-infected tumors, EBV-infected STAD (59.52% vs 17.61%) and HPV-infected HNSC (44.12% vs 16.12%) presented higher fractions of the “Immune-Stimulation” subtype. Fractions of the “Immune-Anergy” subtype were lower in EBV-infected STAD (35.71% vs 74.93%) and HPV-infected HNSC (47.06% vs 78.04%) (Fig. 7A). Kaplan-Meier analysis further demonstrated prognostic significance of TIME subtypes in STAD and HNSC. The “Immune-Exclusion” subtype exhibited the best overall survival (OS) (*P* = 0.026, log-rank test) and progression-free interval (PFI) (*P* = 0.041, log-rank test) in STAD, and the “Immune-Stimulation” subtype showed the best OS (*P* = 0.014, log-rank test) and PFI (*P* = 0.029, log-rank test) in HNSC (Fig. 7B). In contrast, TIME subtypes showed no significant difference in the prognosis of LIHC (Supplementary Fig. S6).

### Construction of the eVIIS pipeline

**Fig. 8:**
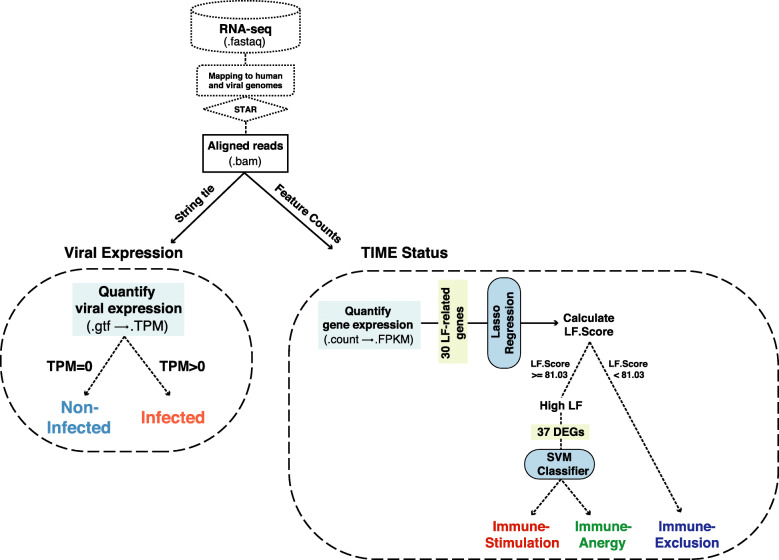
Construction of eVIIS pipeline. The eVIIS pipeline includes several steps: (1) align RNA-seq data to human and viral reference genome sequences using STAR (version <= 2.5); (2) For viral expression detection: use StringTie (version <= 1.2.3) to assemble mapped reads and quantify viral expressions into TPM values. Samples with TPM over zero will be predicted as “Infected”, and samples with TPM equals zero will be predicted as “Nnon-Infected”; (3) For TIME status prediction: use featureCounts (version > = 1.5.0) to obtain read counts and normalize gene expressions into FPKM values. TIME prediction will be made based on the two models (LASSO regression and SVM). For a given sample, eVIIS first calculates its LF.Score using the LASSO regression model based on FPKM values of 30 LF-relevant genes. The sample will be classified as “Immune-Exclusion” if LF.Score < 81.03. Otherwise, it will be further evaluated by the SVM model as “Immune-Stimulation” or “Immune-Anergy”, using FPKM values of the 37 DEGs. The eVIIS pipeline is available at https://github.com/HuangLab-Fudan/eVIIS

We combined the viral sequence detection pipeline and the TIME subtyping workflow to develop an integrated eVIIS pipeline. Given an RNA-seq dataset, eVIIS simultaneously evaluates viral infection and immune status (Fig. 8). eVIIS provides a stepwise or one-step analysis for each sample. Users can choose the appropriate mode for different purposes and different forms of the dataset. Details about the usage of the eVIIS pipeline are described in the README file (https://github.com/HuangLab-Fudan/eVIIS).

For independent validation, we used an extra dataset including 83 human primary gastric tumor tissue samples from the surgical specimen archives from Fudan University Shanghai Cancer Center (FUSCC). The results showed that 4 EBV-infected samples and 12 CMV-infected samples, in which 6 samples (7.23%) were predicted as the “Immune-Stimulation” subtype. The results were consistent with the prevalence of EBV and CMV infections in gastrointestinal tumors observed in the TCGA cohort.

## Discussion

In this study, we designed an NGS-based pipeline to detect 212 types of viral sequences. We obtained a comprehensive landscape of viral expressions of 11,206 tumors of 33 cancer types from TCGA. Of all the infected cancers, stronger virus-cancer associations were observed in CESC, LIHC, LAML, STAD, HNSC and ESCA. And HPV, HBV, EBV and CMV were the most prevalent infection types. Our results are consistent with a similar study that reported HPV infection in 96.55% of tumors (84 out of 87) in CESC, HBV infection in 32.35% of tumors (11 out of 34) in LIHC, and HPV infection in 14.14% of tumors (43 out of 304) in HNSC [11]. However, there’re also some disagreements relating to EBV and CMV infections. For STAD, the study only detected one EBV-infected tumor in STAD (2.3%; 1 out of 43). However, the number is much lower than the number of tumors of the GI.EBV TCGA subtype (7.8%; 30/383) [37]. Furthermore, the occurrence of SV40 and CMV in LAML was controversial in several studies [43–47]. Our results supported the SV40 infection in LAML and we also detected high proportions of CMV infection in LAML. These differences might due to different standards of the definition of viral infections. One of those studies used the expression levels of oncogenic viruses HBV and HPV as referrence and they excluded samples with viral expressions less than 0.5 ppm (p.p.m.) [46]. The purpose is to neglect the trace signals generated by the infiltrated virus-positive lymphocytes instead of the tumor cells themselves [48, 49]. In contrast, we didn’t discard these weak signals. One reason is these weak signals can be caused by technical issues and the exclusion of weak virus-cancer associations might miss out on rare virus-cancer associations [50, 51]. The other reason is that the trace signals coming from infiltrated virus-positive lymphocytes may sensitively reflect the immune status. In a recent study, after filtering low viral expressions, the study showed that the CIBERSORT estimation of absolute immune cell infiltrations was not significantly different between EBV-infected and non-infected tumors in multiple cancers, including STAD [52]. Therefore, they rejected the idea that the detection of EBV is due to infiltrating immune cells and confirmed the active contribution of EBV to STAD. However, the sensitivity of EBV infection to reflect immune status is undercut. We showed no viral infection in eight cancers, including ACC, BRCA, GBM, KICH, PCPG, TGCT, THYM and UVM. The absence of viral infection in BRCA and GBM has been reported previously [11], and our results may offer convincing evidence against viral etiology in the rest tumors.

Viral infections showed different tissue tendencies and their main hosts were highly selective. This could be explained by their different viral receptors that are required during infections. However, viral infections were also detected sporadically in some uncommon hosts. HPV16 was detected in a broad spectrum of cancers including uterus, lung, bladder carcinomas and low-grade gliomas tumors. These have also been reported in previous findings [10, 11, 53]. In our study, HPV-infected BLCA presented as the third most prevalent type of all HPV infections and this is consistent with previous results [52]. Moreover, among different HPV variants, HPV33 ranked the third most prevalent type of HPV infection in HNSC. And a recent study also reported HPV33 in HNSC (*n* = 3). Besides, the study reported HPV6 and HPV45 infections in BLCA. These have also been detected in our results and we additionally detected HPV52 and HPV56. It’s technically hard to discard all the false-positive results of viral infections, but the varied tissue tendencies and expression levels could be used as a reference. For example, HBV-infected LIHC tumors usually harbor high viral expressions. Therefore, in tumors with low HBV viral expressions, they would likely be considered as contaminations or from infected lymphocytes [54], and this has been proved to be a contaminant in KIRC [11]. Similarly, further assessments of low-expressed CMV-infections and SV40-infections in OV will also be needed to exclude contaminations [51]. Compared to this, the uncommon hosts with high levels of viral expressions may represent a special subtype and the diagnostic and therapeutical values should be further explored.

Commonly, viral infections have the potential to cause perturbations in the host genome. In our study, EBV-infected STAD showed decreased CNV and HRD and HPV-infected HNSC exhibited consistently decreased genomic variations. This could partially be explained by the following transcriptional analyses that pathways involving DNA replication, mismatch repair, base excision repair, and nucleotide excision repair in S1 and H3 were upregulated, leading to decreased genomic instability in EBV-infected STAD and HPV-infected HNSC. A recent study reported that HPV-positive HNSC exhibited an almost complete mutual exclusivity with mutations in known drivers such as TP53, CDKN2A and TERT. Such decreased mutation burden and the independence from carcinogenic drivers confirmed the mutation-independent oncogenic and tumorigenic potential of HPV [52].

The impacts of viral infections were different on the host transcriptomes. While the small number and small changes of expression levels of DEGs were seen in HBV-infected LIHC, much greater changes were observed in EBV-infected STAD and HPV-infected HNSC. The common genes that were changed in all types of infections were primarily concerning cell proliferation. This reflects the ability of viral infections to stimulate cell proliferation that leads to tumor development [2]. The clustering results provided more interpretable results that the cell proliferation-relevant DEGs were consistently upregulated in HBV-infected LIHC and HPV-infected HNSC, compared to the inconsistent change in EBV-infected STAD. We showed that different viral infections harbored varied expression levels, with HBV and HPV infections displaying the highest expression levels. This could be interpreted as the greater ability of HBV and HPV to enhance cell proliferation, which leads to higher levels of viral expressions.

Another group of genes that changed commonly were immune-related. These immune-related genes were consistently upregulated in EBV-infected STAD and HPV-infected HNSC. Accordingly, multiple immune cells were increased in the HPV-infected HNSC. This could be supported by a recent study that reported a significant increase in M1 macrophages and T-cells (follicular helper, CD8+ T cells and regulatory T cells) in HPV-positive HNSC [52]. Many immune-stimulating signaling pathways were also upregulated in EBV-infected STAD and HPV-infected HNSC. Of note, the RIG-1 like receptor signaling pathway is primarily responsible for detecting and eliminating viral pathogens. Moreover, immune-suppressive pathways and molecular were also upregulated in the two types of infections. This finding indicates that EBV and HPV infections could elicit effective anti-viral immune responses that were concomitant with immune suppression. It’s worth noting that though immune responses are often aroused by mutation-driven neoantigens, in our study, viral infections were independent of MSI status for most infected tumors exhibited low or intermediate TMB levels. Therefore, the stimulated immune responses might largely be caused by viral infections rather than the intrinsic tumor genomic variations. This result highlights the potential of viral infections being independent markers from MSI-high or TMB.

For anti-PD immunotherapy, TIME is considered as a prerequisite to select appropriate patients [55]. A large number of studies have tried to identify TIME subtypes [56, 57], in which the type displaying high-level tumor-infiltrating leukocytes and stimulated PD pathway was suggested as the optimal one [55]. Since T cells could be functionally impaired and merely serve as bystanders in many tumors with persistent viral infections, the existence of immune cells doesn’t guarantee an effective anti-tumor immune response [58–60]. For this concern, we took both immune cellular infiltrations and their functionality into account to construct the TIME subtyping system model. The model contains three subtypes. The “Immune-Stimulation” subtype showed increased leukocyte infiltration, enhanced cytotoxic activity and immunosuppressive status, especially the increased CD274 (PD-L1) expression level, which could be considered as an appropriate candidate for the anti-PD therapy. EBV-infected STAD and HPV-infected HNSC showed increased fractions of the “Immune-Stimulation” subtype and decreased fractions of the “Immune-Anergy” subtype. Because most EBV- and HPV-infected tumors lacked MSI-high status or high TMB burden and may still have some portion of the “Immune-Stimulation” subtype, highlighting the potential of combing viral infections and immune status to select responders in this group of patients. Recently, a study reported a gastric cancer patient who responded to the anti-PD-L1 drug avelumab. This patient showed no high TMB or MSI-high markers, but the tumor was strongly positive for EBV mRNA [61]. This indicates that viral infection could be a biomarker of immunotherapy to help recognize responders without high TMB or MSI-high markers. In this regard, evaluating viral infection and TIME status could be of great importance to immunotherapy, and the eVIIS pipeline that can simultaneously evaluate viral infection and TIME status could help achieve this goal. Considering the fruitful results from previous studies [10, 11, 52], we did not perform DNA-based fusion site analysis and focused our research on the transcriptome-based detection results. Since viral infections not expressed at the transcript level may be missed out, the results could be further improved by incorporating genome-based results.

## Conclusions

We provided a comprehensive virus-cancer association landscape and revealed different properties of viral infections. EBV-infection and HPV-infection led to decreased genomic variations, significantly altered gene expressions, and effectively triggered anti-viral immune responses in STAD and HNSC. EBV-infection and HPV-infection combined with the TIME subtype could be candidate biomarkers of the immunotherapy in STAD and HNSC, respectively. Finally, the eVIIS pipeline could be a practical tool to facilitate clinical practice and relevant studies.

## Supplementary Information

**Additional file 1: Supplementary** **Fig. S1.** Results of the fuzzy c-means (FCM) clustering. EBV-infected STAD showed 3 gene clusters (S1, S2, S3), HBV-infected LIHC showed 4 gene clusters (L1, L2, L3, L4) and HPV-infected HNSC showed 4 gene clusters (H1, H2, H3, H4). **Supplementary Fig. S2.** Comparisons of GO enrichment analysis of DEGs in each cluster, in which left side denotes down-regulated DEGs and right side for up-regulated DEGs. The bubble size represents enriched genes in each GO term and color indicates adjust.*P* value. **Supplementary Fig. S3.** CD8 T cells infiltration in all EBV- and HPV-associated cancers. The red triangles and grey rounds represent virus-positive and virus-negative cases respectively. **Supplementary Fig. S4.** Correlation between CD8 T cells infiltration and EBV expression in all EBV-associated cancers. The different colored points stand for the corresponding cancer types. **Supplementary Fig. S5.** Comparisons of Neoantigen, TCR and BCR between infected cases and non-infected cases in STAD, LIHC and HNSC. **Supplementary Fig. S6.** Survival curves of OS and PFI outcomes of TIME subtypes in LIHC. **Supplementary Fig. S7.** ROC-curve of LF.Score classifier in training (3865 samples),validation1 (2931 samples) and validation2 (2911 samples) cohorts; the accuracy of the SVM diagnosis in two random subsampling cohorts were labeled. **Supplementary Fig. S8.** 10 cross-validation curve (red dotted line), and upper and lower standard deviation curves along the λ sequence (error bars). We determined lambda.1se (0.00673) as the optimal λ, which gives the most regularized model such that error is within one standard error of the minimum. **Supplementary Fig. S9.** ROC-curve and confusion table of SVM classifier in training (*n* = 1200) and validation (*n* = 513) cohorts. **Supplementary Fig. S10.** Correlation analysis between TIME subtypes and TIDE. **Supplementary Fig. S11**. Correlation analysis between TIME subtypes and IFNG score.

**Additional file 2: Supplementary Table 1.** TCGA Dataset.

**Additional file 3: Supplementary Table 2.** Comparisons of genomic features between infected and non-infected tumors.

**Additional file 4: Supplementary Table 3.** Information of 76 immune-related genes.

**Additional file 5: Supplementary Table 4.** Gender, Race and Age analyses.

**Additional file 6: Supplementary Table 5.** TIDE and TIME information for TCGA SKCM dataset.

## Data Availability

The datasets generated and/or analysed during the current study are available in the https://gdc.cancer.gov/access-data/gdc-data-portal. The FUSCC dataset is not shown but is available upon reasonable request. The eVIIS pipeline is available at https://github.com/HuangLab-Fudan/eVIIS.

## Abbreviations

ACC: Adrenocortical carcinoma
BLCA: Bladder Urothelial Carcinoma
BRCA: Breast invasive carcinoma
CESC: Cervical squamous cell carcinoma and endocervical adenocarcinoma
CHOL: Cholangiocarcinoma
COAD: Colon adenocarcinoma
DLBC: Lymphoid Neoplasm Diffuse Large B-cell Lymphoma
ESCA: Esophageal carcinoma
GBM: Glioblastoma multiforme
HNSC: Head and Neck squamous cell carcinoma
KICH: Kidney Chromophobe
KIRC: Kidney renal clear cell carcinoma
KIRP: Kidney renal papillary cell carcinoma
LAML: Acute Myeloid Leukemia
LGG: Brain Lower Grade Glioma
LIHC: Liver hepatocellular carcinoma
LUAD: Lung adenocarcinoma
LUSC: Lung squamous cell carcinoma
MESO: Mesothelioma
OV: Ovarian serous cystadenocarcinoma
PAAD: Pancreatic adenocarcinoma
PCPG: Pheochromocytoma and Paraganglioma
PRAD: Prostate adenocarcinoma
READ: Rectum adenocarcinoma
SARC: Sarcoma
SKCM: Skin Cutaneous Melanoma
STAD: Stomach adenocarcinoma
TGCT: Testicular Germ Cell Tumors
THCA: Thyroid carcinoma
THYM: Thymoma
UCEC: Uterine Corpus Endometrial Carcinoma
UCS: Uterine carcinosarcoma
UVM: Uveal Melanoma
AUC: Area under curve
CMV: Cytomegalovirus
CNV: Copy number variation
DEG: Differentially expressed gene
EBV: Epstein-Barr virus
FCM: Fuzzy c-means
FPKM: Fragments per kilobase million
GDC: Genomic Data Commons
GSVA: Gene set variation analysis
HBV: Hepatitis B virus
HCV: Hepatitis C virus
HIV: Human Immunodeficiency virus
HPV: Human Papilloma virus
HRD: Homologous recombination defects
ITH: Intratumor heterogeneity
KSHV: Kaposi’s sarcoma-associated herpes virus
LF: Leukocyte fraction
MCV: Merkel cell polyomavirus
MMR: Mismatch repair
MSI: Microsatellite instability
MSigDB: Molecular Signatures Database
NGS: Next-generation sequencing
ROC: Receiver operating characteristic curve
SNV: Single nucleotide variant
SV40: Simian virus 40
SVM: Support vector machine
TCGA: The Cancer Genome Atlas
TCR: T cell receptor
TIME: Tumor immune microenvironment
TMB: Tumor mutational burden
TPM: Transcripts per kilobase million
OS: Overall survival
PFI: Progression-free interval
