# Supplementary material for: The viral expression and immune status in human cancers and insights into novel biomarkers of immunotherapy
Source: BMC Cancer. 2021 Nov 5;21:1183. doi: 10.1186/s12885-021-08871-9 (PMC8571886; doi:10.1186/s12885-021-08871-9)
Supplement: Supplementary file 1 — Additional file 1: Supplementary Fig. S1. Results of the fuzzy c-means (FCM) clustering. EBV-infected STAD showed 3 gene clusters (S1, S2, S3), HBV-infected LIHC showed 4 gene clusters (L1, L2, L3, L4) and HPV-infected HNSC showed 4 gene clusters (H1, H2, H3, H4). Supplementary Fig. S2. Comparisons of GO enrichment analysis of DEGs in each cluster, in which left side denotes down-regulated DEGs and right side for up-regulated DEGs. The bubble size represents enriched genes in each GO term and color indicates adjust.P value. Supplementary Fig. S3. CD8 T cells infiltration in all EBV- and HPV-associated cancers. The red triangles and grey rounds represent virus-positive and virus-negative cases respectively. Supplementary Fig. S4. Correlation between CD8 T cells infiltration and EBV expression in all EBV-associated cancers. The different colored points stand for the corresponding cancer types. Supplementary Fig. S5. Comparisons of Neoantigen, TCR and BCR between infected cases and non-infected cases in STAD, LIHC and HNSC. Supplementary Fig. S6. Survival curves of OS and PFI outcomes of TIME subtypes in LIHC. Supplementary Fig. S7. ROC-curve of LF.Score classifier in training (3865 samples),validation1 (2931 samples) and validation2 (2911 samples) cohorts; the accuracy of the SVM diagnosis in two random subsampling cohorts were labeled. Supplementary Fig. S8. 10 cross-validation curve (red dotted line), and upper and lower standard deviation curves along the λ sequence (error bars). We determined lambda.1se (0.00673) as the optimal λ, which gives the most regularized model such that error is within one standard error of the minimum. Supplementary Fig. S9. ROC-curve and confusion table of SVM classifier in training (n = 1200) and validation (n = 513) cohorts. Supplementary Fig. S10. Correlation analysis between TIME subtypes and TIDE. Supplementary Fig. S11. Correlation analysis between TIME subtypes and IFNG score. [file 12885_2021_8871_MOESM1_ESM.doc]

**The viral expression and immune status in human cancers and insights into novel biomarkers of immunotherapy**

Siyuan Chen^1,3,4^, Hongyan Lai^2,3,4^, Jingjing Zhao^2^, Bing Chen^1,3^, Yan Li^2^, Yuchen Li^2^, Qin Li^2^, Qiupeng Zheng^2^, Shenglin Huang^2,3, *^, Xiaodong Zhu^1,3, *^

#

^1^Department of Medical Oncology, Fudan University Shanghai Cancer Center, Fudan University, Shanghai, 200032, China

^2^Fudan University Shanghai Cancer Center, Key Laboratory of Medical Epigenetics and Metabolism, Institutes of Biomedical Sciences, Fudan University, Shanghai 200032, China

^3^Department of Oncology, Shanghai Medical College, Fudan University, Shanghai, China

^4^These authors contributed equally to this work

**Corresponding authors:** Xiaodong Zhu, Ph.D., xddr001@163.com; Department of Medical Oncology, Fudan University Shanghai Cancer Center, Fudan University, Shanghai, 200032, China. Tel.: 86-21-64175590*88907; Fax: 86-21-64430366. Or Shenglin Huang, Ph.D., [slhuang@fudan.edu.cn](mailto:slhuang@fudan.edu.cn); Fudan University Shanghai Cancer Center and Institutes of Biomedical Sciences, Fudan University, 270 Dong An Rd., Shanghai 200032, China. Tel.: 86-21-34777580; Fax: 86-21-64172585.

Supplementary Material

**Supplementary Fig.S1-S9**


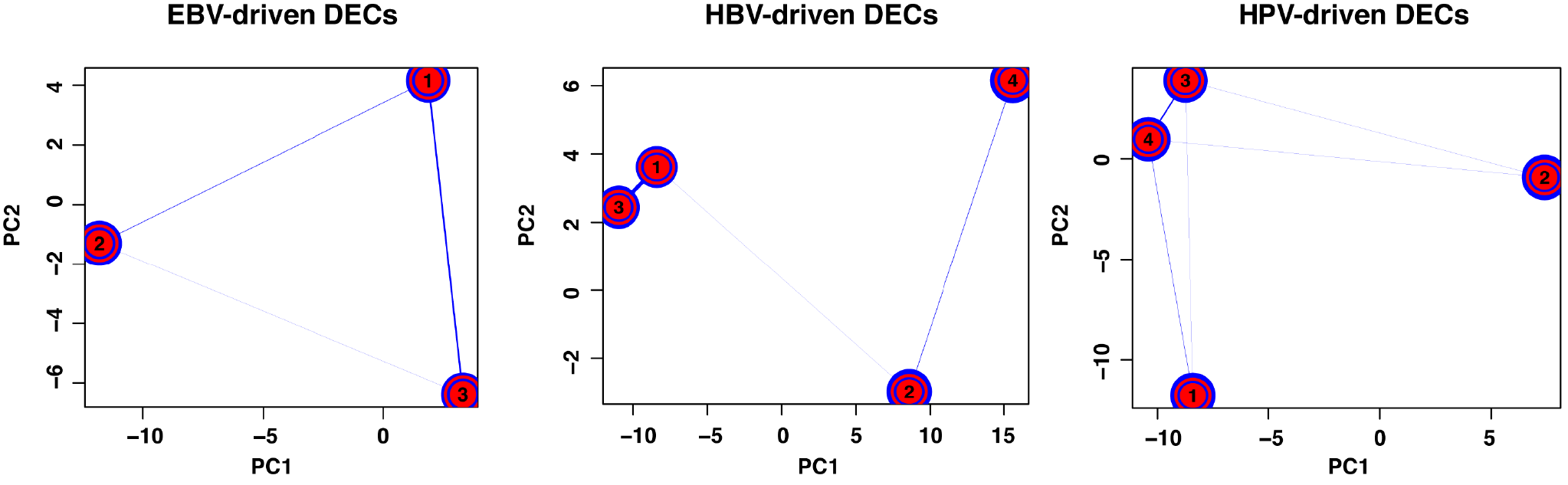


**Supplementary Fig. S1** Results of the fuzzy c-means (FCM) clustering. EBV-infected STAD showed 3 gene clusters (S1, S2, S3), HBV-infected LIHC showed 4 gene clusters (L1, L2, L3, L4) and HPV-infected HNSC showed 4 gene clusters (H1, H2, H3, H4).


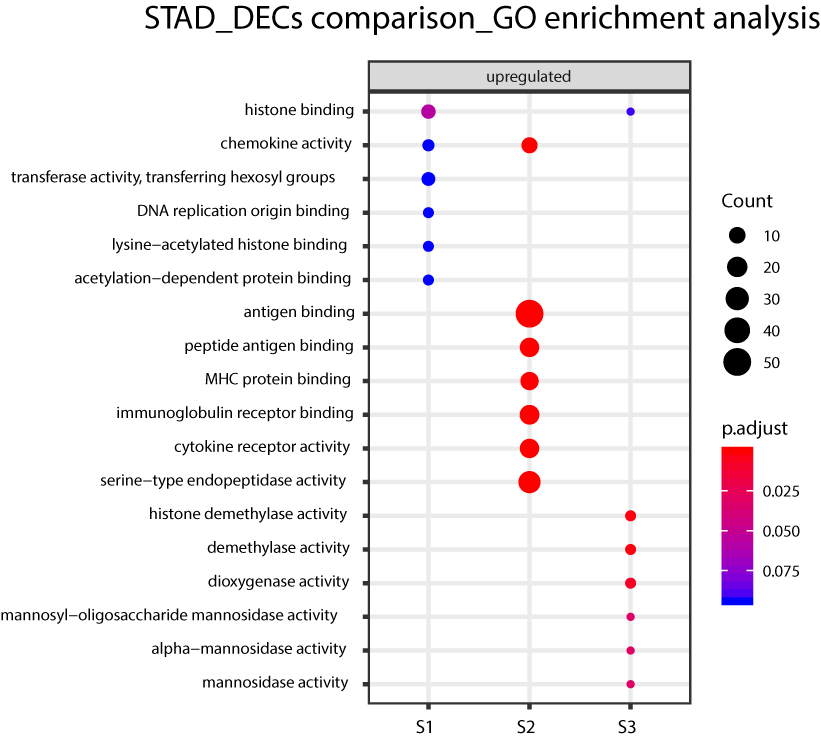


**
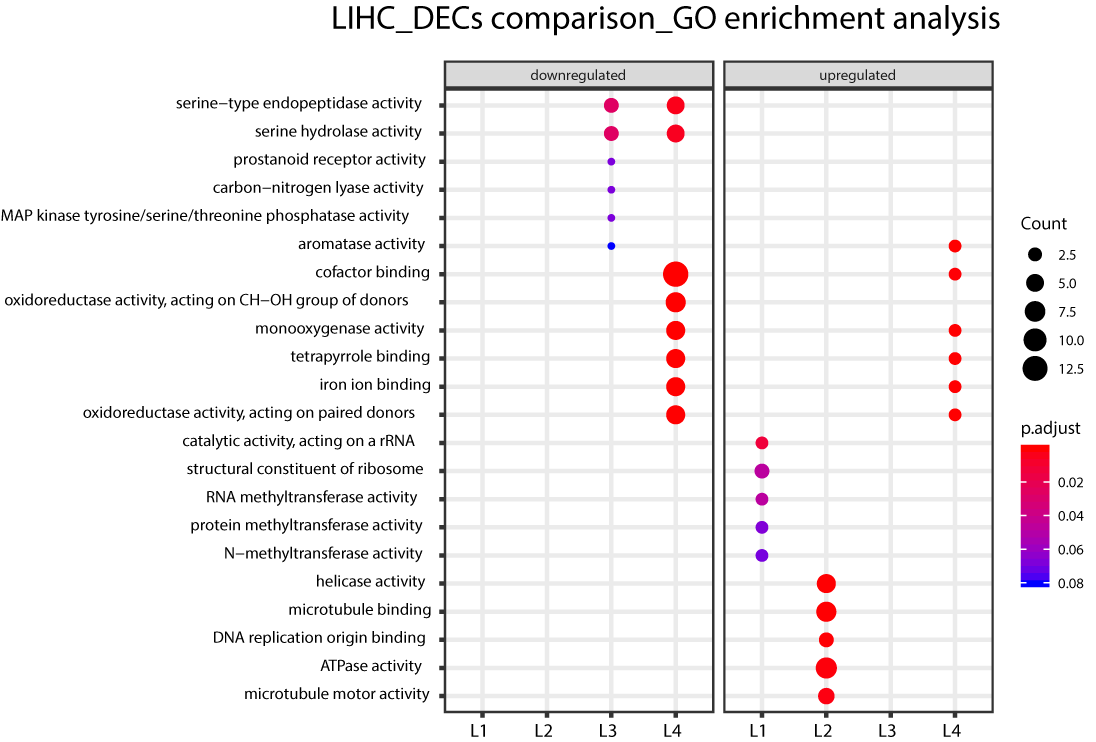
**


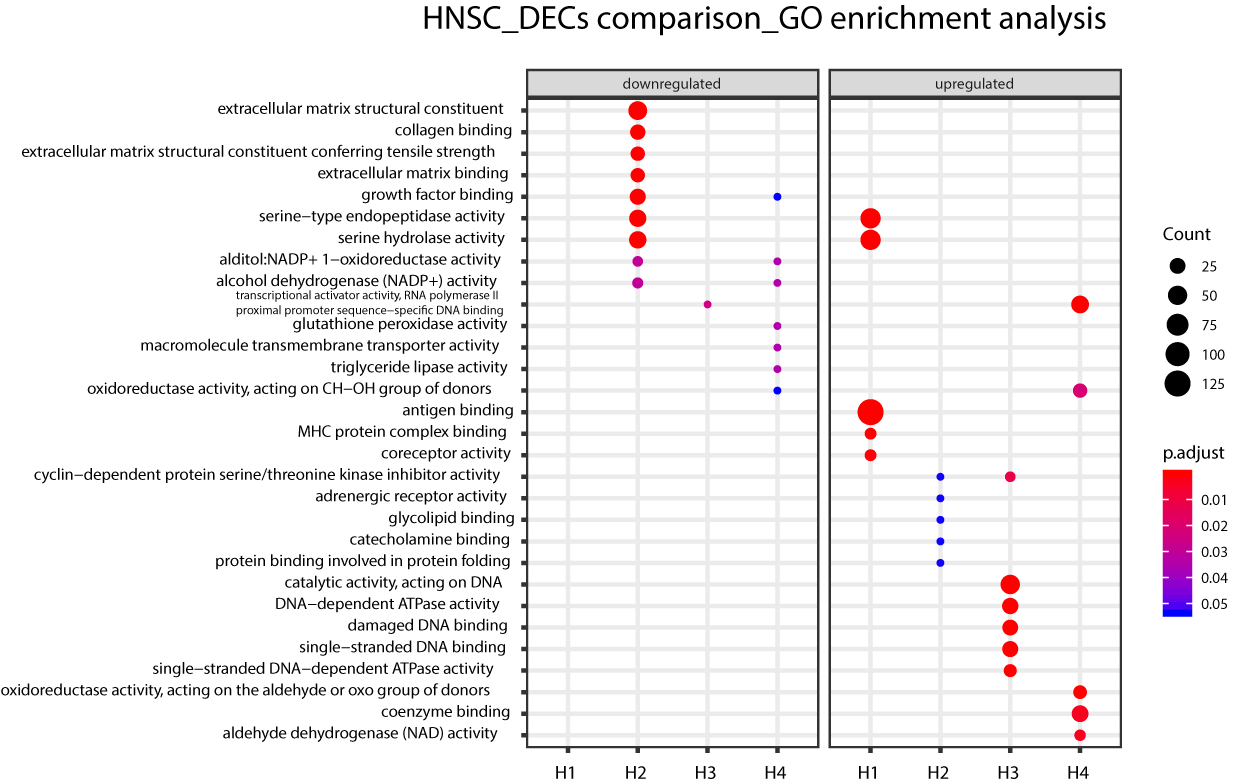


**Supplementary Fig. S2** Comparisons of GO enrichment analysis of DEGs in each cluster, in which left side denotes down-regulated DEGs and right side for up-regulated DEGs. The bubble size represents enriched genes in each GO term and color indicates adjust.P value.


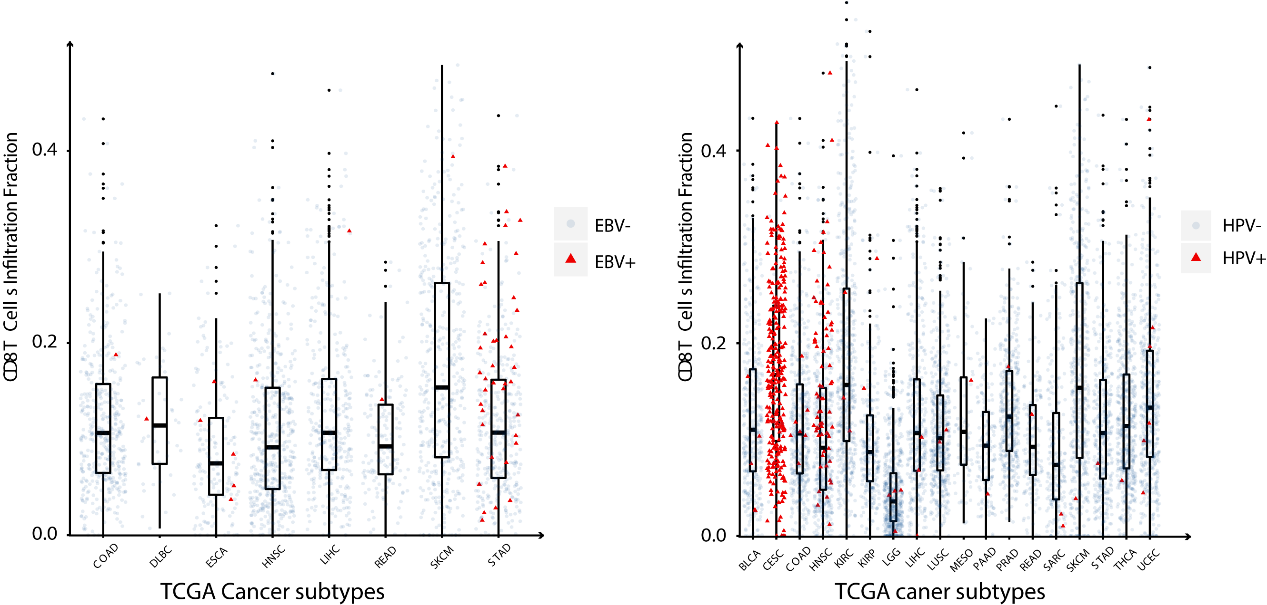


**Supplementary Fig. S3** CD8 T cells infiltration in all EBV- and HPV-associated cancers. The red triangles and grey rounds represent virus-positive and virus-negative cases respectively.


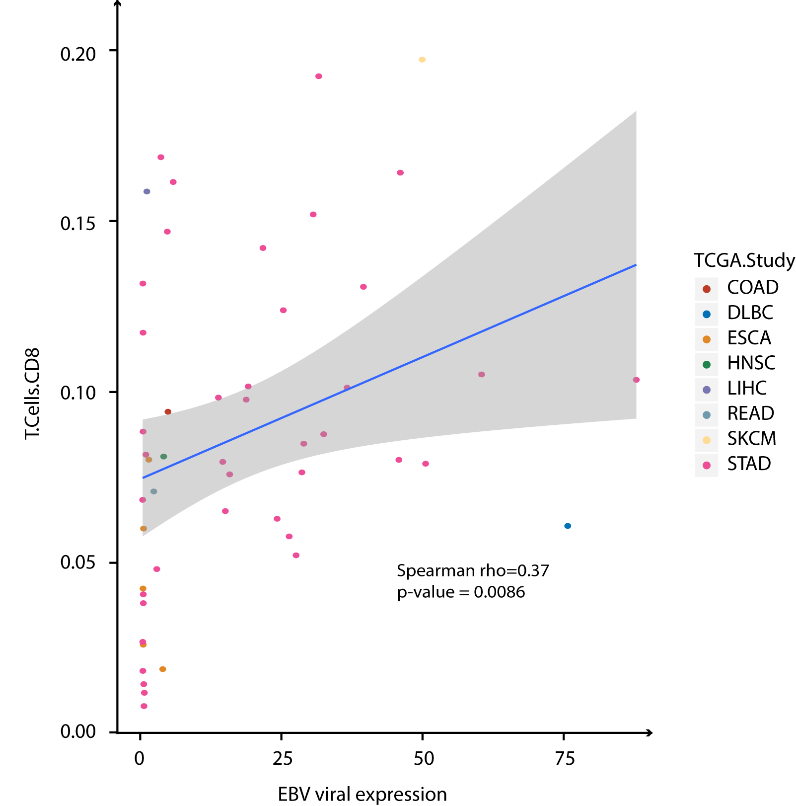


**Supplementary Fig. S4** Correlation between CD8 T cells infiltration and EBV expression in all EBV-associated cancers. The different colored points stand for the corresponding cancer types.

**
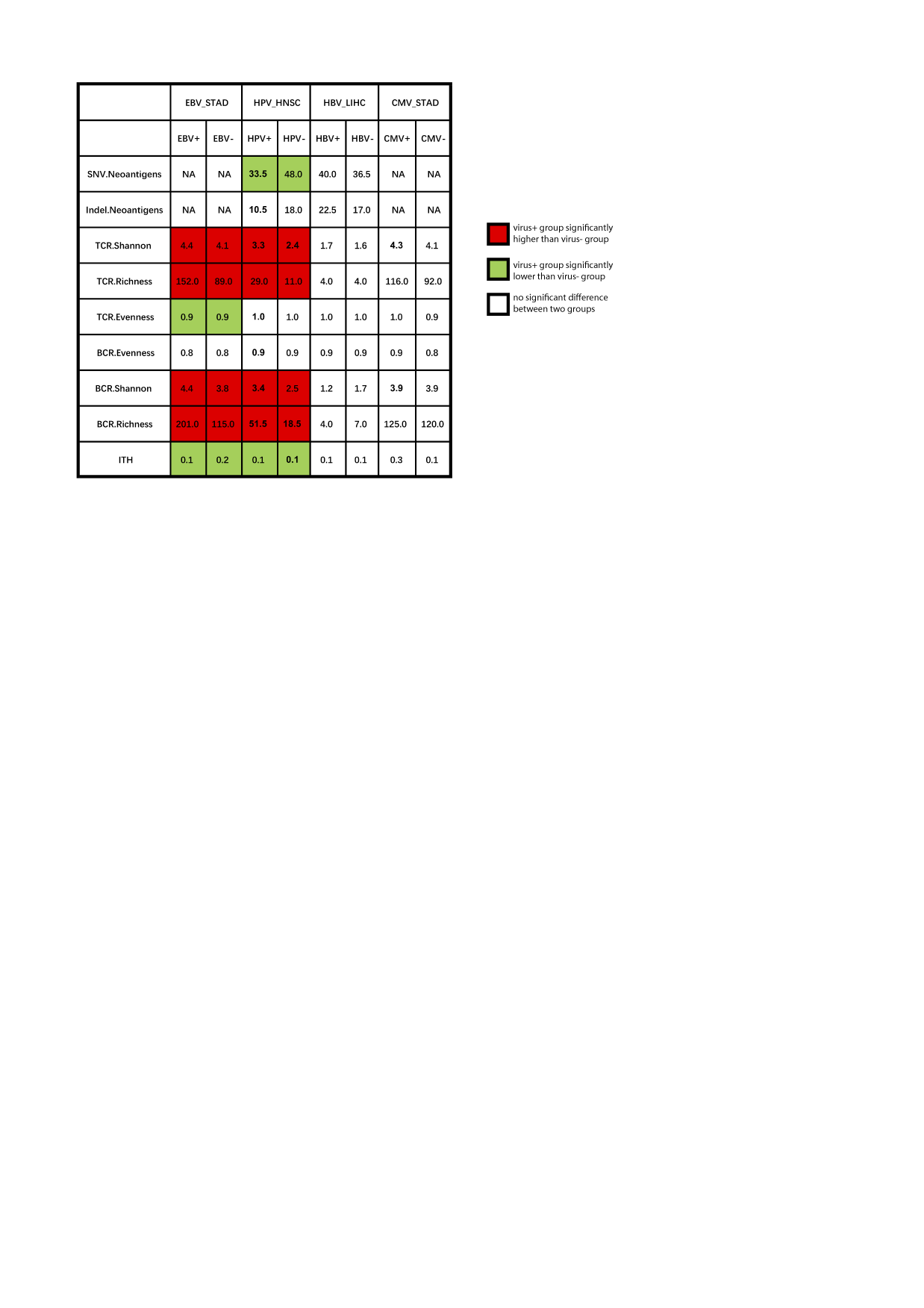
**

**Supplementary Fig. S5** Comparisons of Neoantigen, TCR and BCR between infected cases and non-infected cases in STAD, LIHC and HNSC.


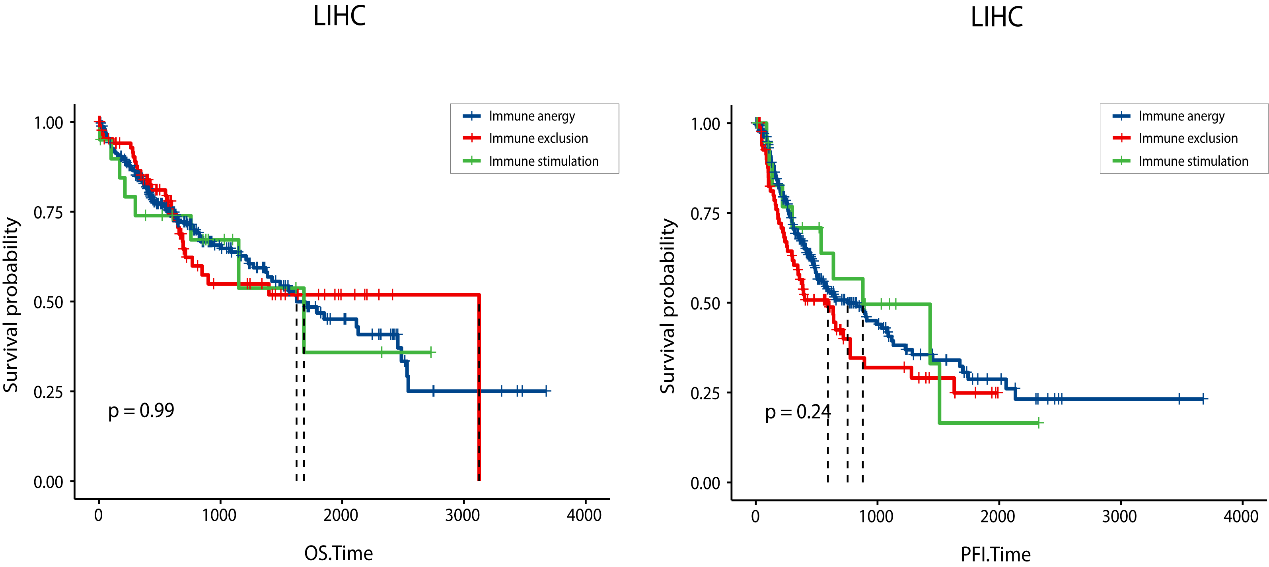


**Supplementary Fig. S6** Survival curves of OS and PFI outcomes of TIME subtypes in LIHC.

**
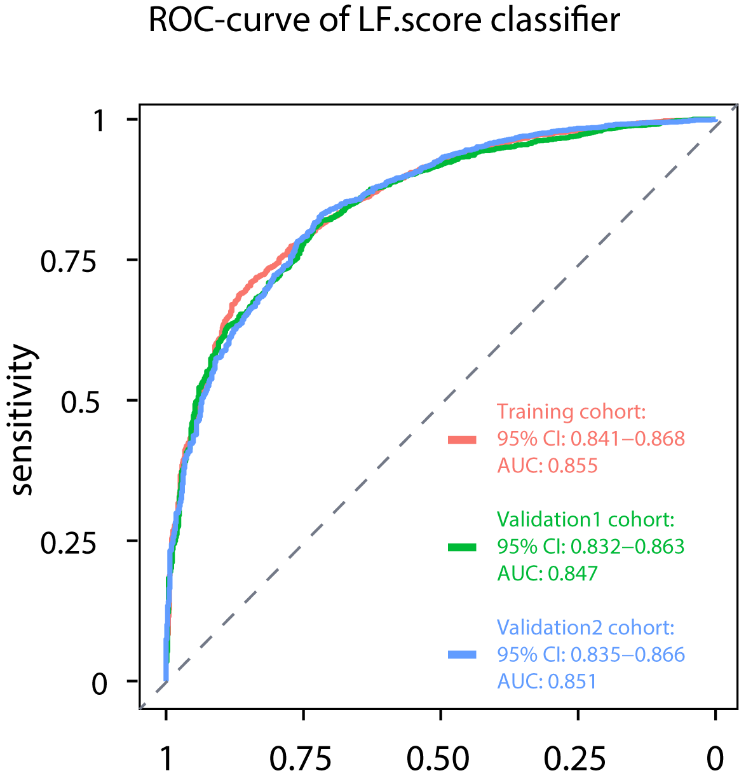
**

**Supplementary Fig. S7** ROC-curve of LF.Score classifier in training (3865 samples),validation1 (2931 samples) and validation2 (2911 samples) cohorts; the accuracy of the SVM diagnosis in two random subsampling cohorts were labeled.


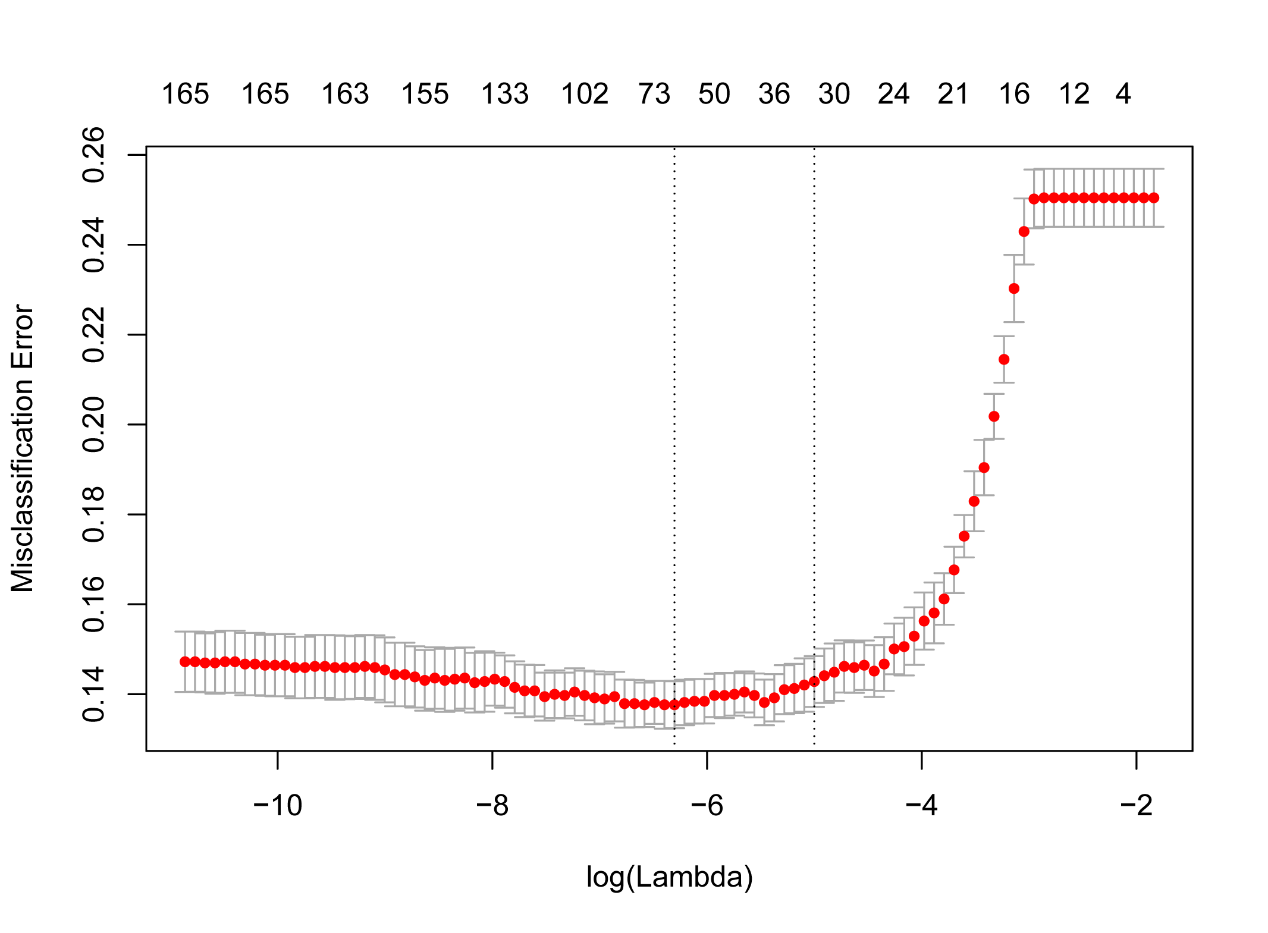


**Supplementary Fig. S8** 10 cross-validation curve (red dotted line), and upper and lower standard deviation curves along the λ sequence (error bars). We determined lambda.1se (0.00673) as the optimal λ, which gives the most regularized model such that error is within one standard error of the minimum.


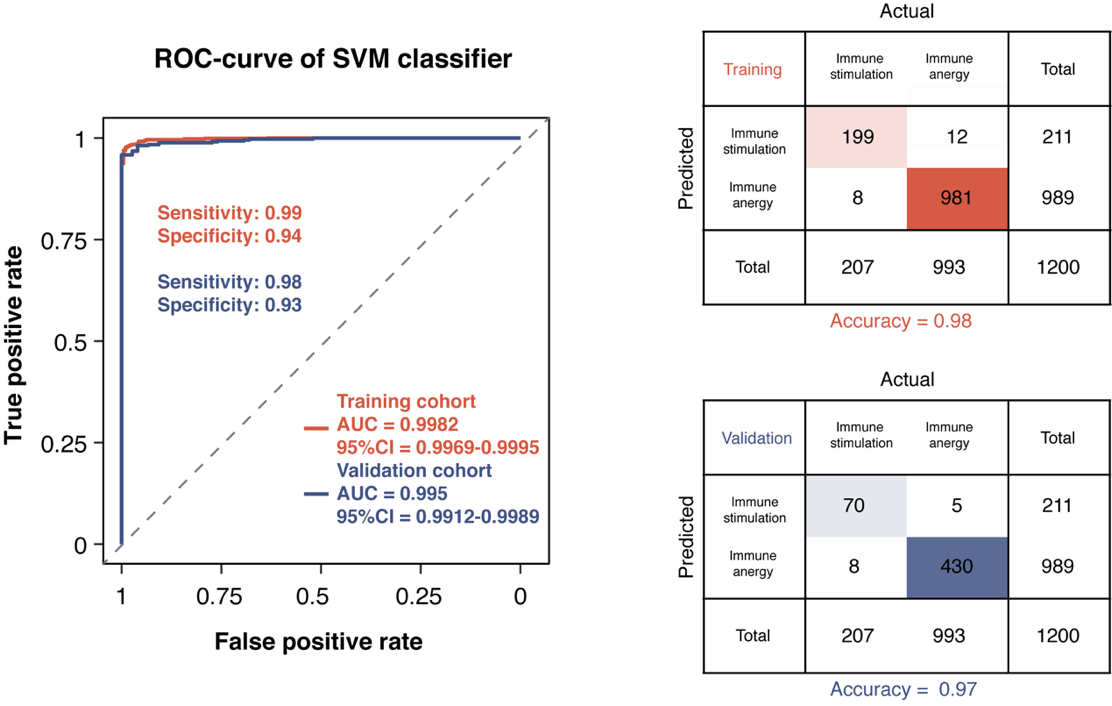


**Supplementary Fig. S9** ROC-curve and confusion table of SVM classifier in training (n=1200) and validation (n=513) cohorts.


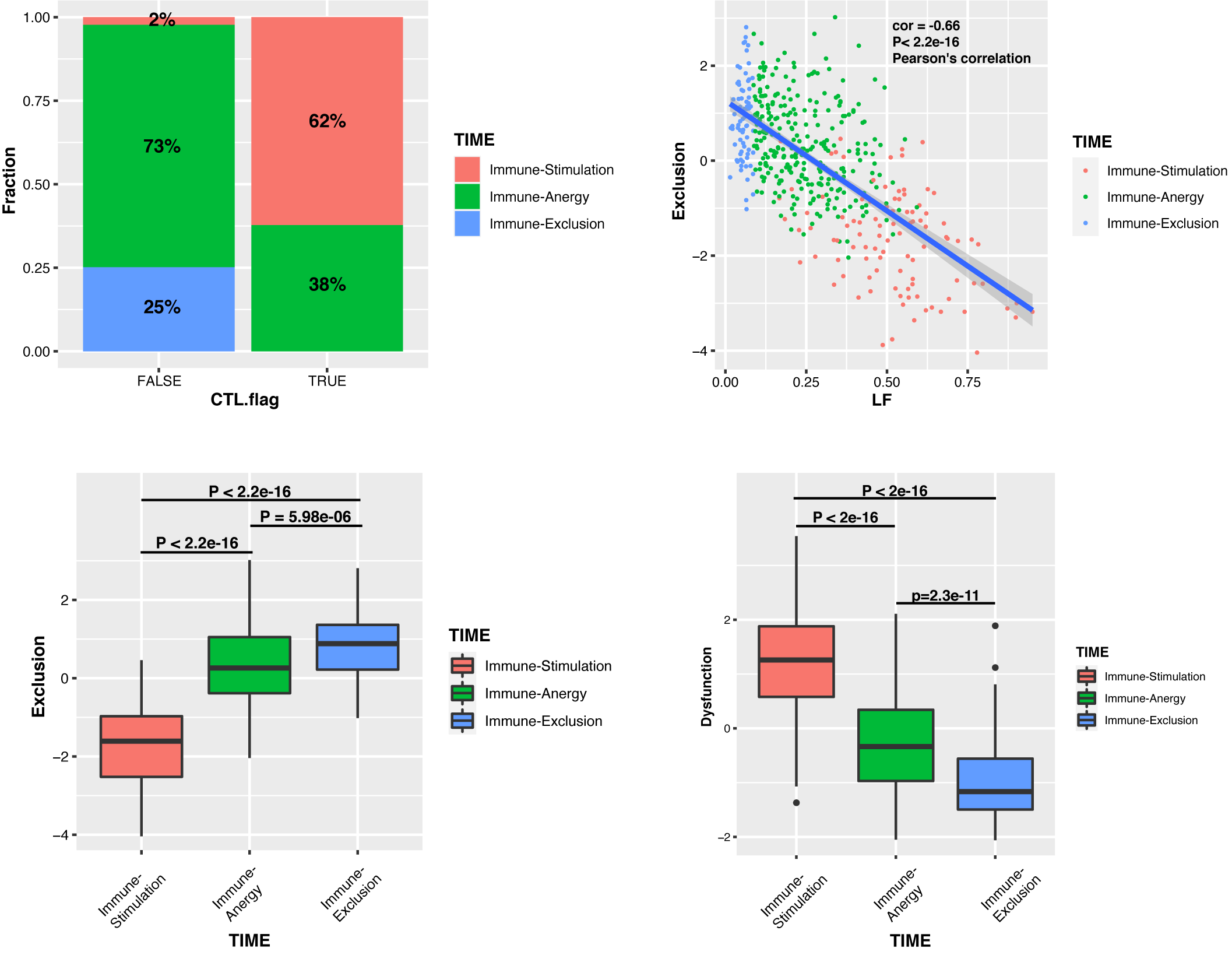


**Supplementary Fig. S10** Correlation analysis between TIME subtypes and TIDE


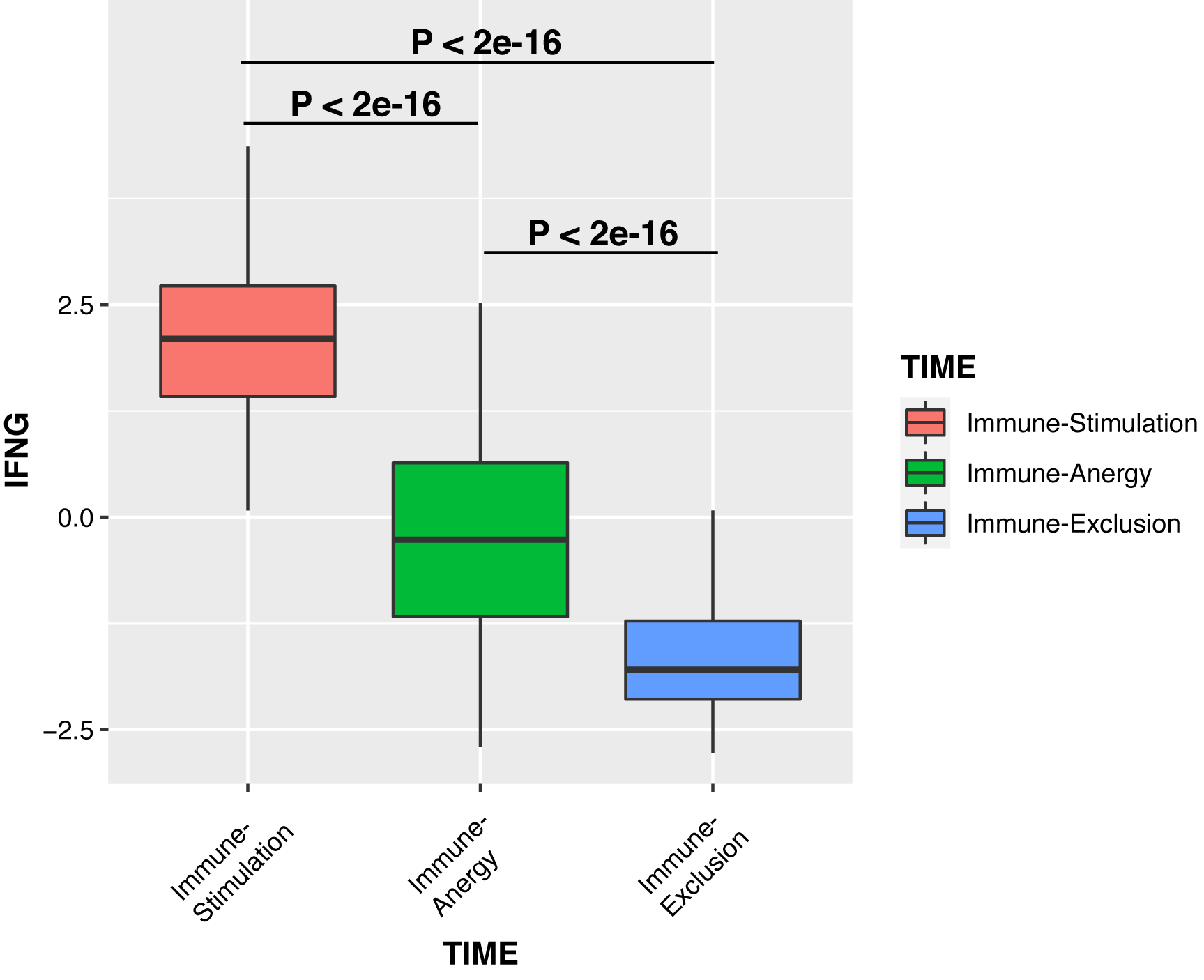


**Supplementary Fig. S11** Correlation analysis between TIME subtypes and IFNG score
